# Genome Analysis of Minibacterium massiliensis Highlights the Convergent Evolution of Water-Living Bacteria

**DOI:** 10.1371/journal.pgen.0030138

**Published:** 2007-08-24

**Authors:** Stéphane Audic, Catherine Robert, Bernard Campagna, Hugues Parinello, Jean-Michel Claverie, Didier Raoult, Michel Drancourt

**Affiliations:** 1 Structural and Genomic Information Laboratory, Institute for Structural Biology and Microbiology, Marseille, France; 2 CNRS, UPR2589, Marseille, France; 3 Unité des Rickettsies, Faculté de Médecine, Université de la Méditerranée, Marseille, France; 4 CNRS, UMR6020, Marseille, France; Department of Energy Joint Genome Institute, United States of America

## Abstract

Filtration usually eliminates water-living bacteria. Here, we report on the complete genome sequence of Minibacterium massiliensis, a β-proteobacteria that was recovered from 0.22-μm filtered water used for patients in the hospital. The unexpectedly large 4,110,251-nucleotide genome sequence of M. massiliensis was determined using the traditional shotgun sequencing approach. Bioinformatic analyses shows that the M. massiliensis genome sequence illustrates characteristic features of water-living bacteria, including overrepresentation of genes encoding transporters and transcription regulators. Phylogenomic analysis based on the gene content of available bacterial genome sequences displays a congruent evolution of water-living bacteria from various taxonomic origins, principally for genes involved in energy production and conversion, cell division, chromosome partitioning, and lipid metabolism. This phylogenomic clustering partially results from lateral gene transfer, which appears to be more frequent in water than in other environments. The M. massiliensis genome analyses strongly suggest that water-living bacteria are a common source for genes involved in heavy-metal resistance, antibiotics resistance, and virulence factors.

## Author Summary

Microorganisms are ubiquitous, found in environments including humans and animals, air, soil, and water, even in extreme conditions. Indeed, we isolated an emerging small bacterium M. massiliensis in hemodialysis water despite microbiological control by filtration and chemicals. Its very small size allowed this bacterium to pass through filters. Decoding of its genome revealed the presence of numerous so-called heavy-metal resistance genes encoding protection against chemicals. The genome also encodes virulence factors and antibiotic resistances. Study of M. massiliensis gene content revealed that it shares many genes with other bacteria in its β-proteobacteria family, but also with many other water-living bacteria from other families. Comparison of the M. massiliensis genome with other completely sequenced genomes indicated that a high fraction of genes (17%) had closest neighbors in water-living bacteria from other families. Such lateral gene transfer was further generalized to all water-living bacteria, which mutualize a higher fraction of their genome than bacteria living in other environments. Water is a privileged ecosystem for the exchange of bacterial genes and the emergence of new combinations of virulence and resistance. As new technologies increase the contact of humans with water, its use for medical and recreational usages has to be thoroughly controlled.

## Introduction

**Figure 1:**
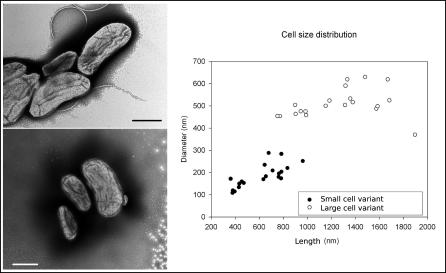
Transmission Electron Microscopy Featuring SCV and LCV of M. massiliensis LCV measured 1,259 + 329 × 510 + 65 nm and SCV measured 615 + 185 × 185 + 52 nm. The distribution of length and diameter of organisms in the M. massiliensis cell population is also shown. (Bar = 500nm)

Industries and health care centers produce ultrapure water (UPW) [1]. It is a complex multi-stage process incorporating pretreatment and polishing stages to remove organic and inorganic compounds and involves filtration as a key step. Some groundwater-borne β- and γ-proteobacteria can grow in the extreme UPW environment [1,2]. Routine microbiological survey of UPW in hemodialysis units yielded a hitherto undescribed filterable and motile β-proteobacteria species herein referred to as M. massiliensis gen. nov. sp. nov. (Table S1). This new organism was regularly isolated over a 7.5-mo period before it was eradicated by repairing the UPW production and distribution system. It exhibited small-cell and large-cell variants (Figure 1). Its close relationship with other filterable freshwater-borne and soil β-proteobacteria was indicated by 16S rDNA–based phylogeny [3–5], although the 16S rDNA sequence exhibits only 90% identity to that of the closest sequenced species (Ralstonia spp., *Burkolderia* spp*.,* and Bordetella spp.). Because of the potential threat represented by an unknown filterable bacteria found in close physical proximity to patients' blood, we sequenced the M. massiliensis genome in order to identify its gene content and compare it to the available genomes of freshwater-borne bacteria.

## Results

### General Genome Features

**Figure 2:**
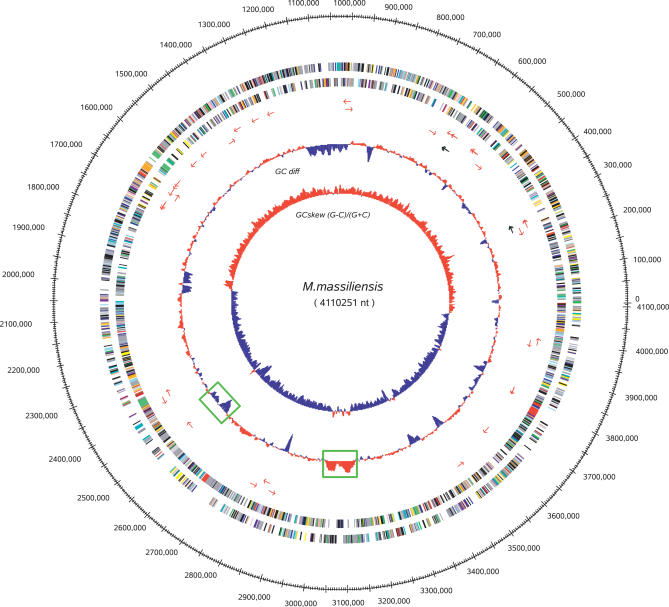
Map of the M. massiliensis Chromosome The following features are displayed (from the outside in): position along the genome, protein-coding genes along both strands colored according to COG categories, tRNA genes as red arrows, rRNA genes as black arrows, the windowed difference of GC% with respect to the average, and the GC skew (G − C)/(G + C), with positive values in red and negative values in blue. Two regions of phage insertion are indicated by green boxes.

The M. massiliensis genome comprises one circular chromosome of 4,110,251 bp with an average 54.22% G + C content (Figure 2), whereas its closest relatives Ralstonia eutropha and Ralstonia solanacearum comprise several large replicons [6]. We predicted 3,697 uniformly distributed protein-coding ORFs that cover 89.07% of the genome and have an average length of 993 bp. No functional attribute could be assigned to 25% of the predicted ORF products, a proportion similar to that found in most newly sequenced bacterial genomes. There are 46 tRNA genes representing all 20 amino acids, one tmRNA, and two rRNA operons identical in their coding regions. The likely origin of replication was identified based on G + C skew and the position of DnaA (mma0001), DnaN(mma0002), and GyrB (mma0003) [7]. Two regions of phage insertion with an atypical G + C content were detected [8]. Electron microscopy observation indicated that M. massiliensis organisms present two subpopulations. Small-cell variants (SCV) average 185 nm × 615 nm (V_SCV_ = 11.0 × 10^−3^ μm^3^, S_SCV_ = 0.29 μm^2^) and large-cell variants (LCV) average 510 nm × 1,259 nm (V_LCV_ = 171 × 10^−3^ μm^3^, S_LCV_ =1.68 μm^2^). SCV were significantly more elongated (length/diameter ratio ≅ 3.3) than LCV (length/diameter ratio ≅ 2.5) (*t*-test, *p* < 0.0005) (Figure 1). The division cell wall *(dcw)* cluster comprised PBP3 (FtsI, mma3021) and FtsW (mma3016) and exhibited the organization characteristic of rod-shaped bacteria [9]. The organization of the rod shape–determining operon encoding MreB (mma0196), MreC (mma0197), MreD (mma0198), PBP2 (MrdA, mma0199), and RodA (MrdB,mma0200) was of a β-proteobacteria type, identical to that found in Nitrosomonas europaea, Bordetella spp. and Burkolderia spp. except for the presence of the rare lipoprotein A RlpA (mma0201). M. massiliensis uniquely comprises three copies of the chromosome-partitioning protein ParA, one of which (ParA1, mma0164) is most closely related but not orthologous to that of the spore-forming Bacillus spp. and Lactobacillus spp. *M*. *massiliensis* was motile due to a single polar flagella. The flagellar cluster consists of one uninterrupted 36-ORF operon (FlgA-N, FliC-T, FlaG, mma1415–1450) and two motor proteins MotAB (mma2081/2082) flanking the FlhABF, FliA, FleN operon (mma2083–2087).

### Transport and Metabolism

Gene content analyses of M. massiliensis revealed that 432/3697 genes (12%) encode transporters (Table S2). This transport capacity is much larger than that in any of the 179 bacteria listed in http://www.membranetransport.org/ [10], which have an average fraction of transport genes of 5.5% ± 1.7%. The M. massiliensis genome encodes a particularly large number of genes for the transport of ions, amino acids, and sugars. This high transport capacity is in contrast with the constrained metabolism of *M. massiliensis,* illustrated by the lack of a gene encoding Glk, a glucokinase involved in the metabolism of unphosphorylated intracellular glucose; this enzyme is widespread, as it is present in 200/282 bacteria and 14/19 β-proteobacteria in the Kegg database [11]. Although pathways for the synthesis of purines, pyrimidines, and amino acids are identified in M. massiliensis, the choice of enzymatic route appears to be limited with respect to what is observed in *R. solanaceraum,* as illustrated by purine metabolism (Figure S1). In particular, the phosphorylation capacities of sugars and nucleotides are strongly reduced.

### Discrepancies between Taxonomy and Gene Content

**Figure 3:**
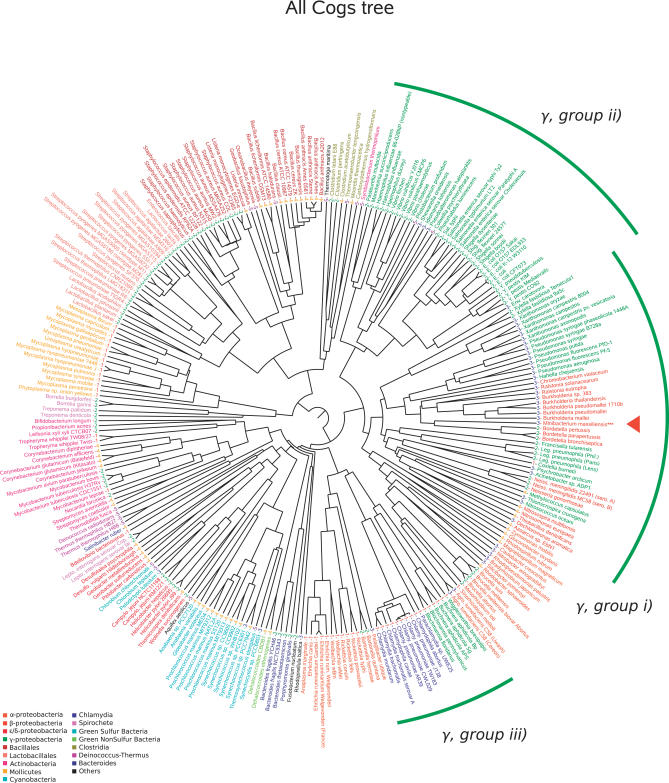
All COG Phylogenomic Representations Organisms are colored according to taxonomy. The class membership of the organisms is also indicated: (1) obligate intracellular bacteria including endosymbionts, (2) pathogens and host-associated bacteria (3) waterborne free-living bacteria, (4) nonwaterborne, free-living bacteria, and (5) extremophiles. The position of M. massiliensis is indicated by a red triangle in the tree.

Comparison of the M. massiliensis ORFs with those of other organisms in the Kegg database [11] revealed that 620 and 429 of the 3,697 ORFs have their closest homologs in the genomes of R. eutropha and R. solanacearum, respectively. To identify relatives of M. massiliensis in terme of gene content, we examined the distribution of clusters of orthologous groups genes (COG) [12] among bacterial genomes classified by lifestyle as follows: (1) obligate intracellular bacteria including endosymbionts and pathogens, (2) pathogens and host-associated bacteria, (3) water-living bacteria, (4) nonwaterborne, free-living bacteria, and (5) extremophiles [13]. Representing the presence or absence of each COG in an organism as a vector, we computed a phylogenomic tree from the matrix of interorganism distances (see Materials and Methods). This phylogenomic analysis yielded a tree grossly similar to that derived from the 16S rDNA gene sequence, grouping together bacteria from the same taxon (Figure 3). However, in our analysis, γ-proteobacteria appeared to be divided into three groups: (i) environmental γ-proteobacteria clustered with environmental β-proteobacteria, including *M. massiliensis;* (ii) enteric γ-proteobacteria forming an unique clade along with *Vibrio* species; and (iii) intracellular γ-proteobacteria clustered with intracellular α-proteobacteria and Chlamydia spp., although the lattermost cluster, which groups small-sized genomes, could be artefactual. In this tree, M. massiliensis clustered with other microorganisms according to their waterborne lifestyle (category 3) rather than according to the 16S rDNA–based phylogeny. COG-specific trees were examined to determine which categories of genes displayed a similar pattern. The same grouping of water-living bacteria was particularly apparent when focusing on genes belonging to the following three functional categories: C-COG (energy production and conversion), D-COG (cell division and chromosome partitioning), and I-COG (lipid metabolism) (Figure S2).

### Gene Exchange

**Figure 4:**
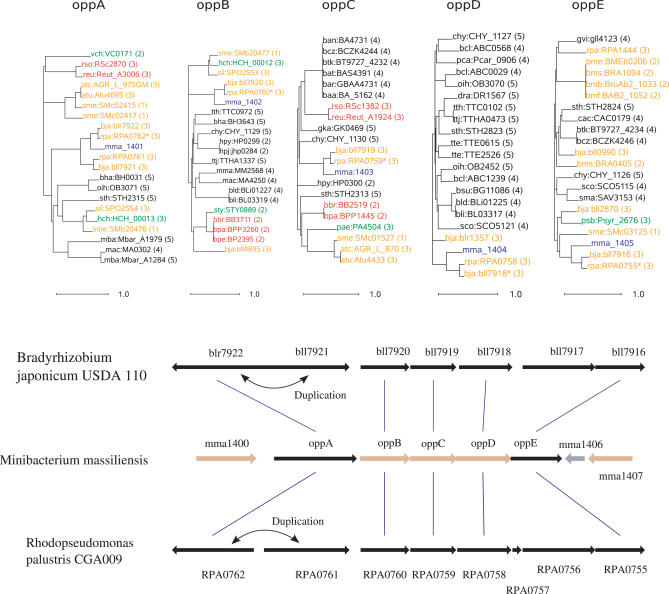
En-Bloc Gene Transfer Phylogenetic trees for five consecutive genes in M. massiliensis illustrating lateral gene transfer with α-proteobacteria. Genes are labeled according to their names in the Kegg database, followed by the environmental category of the organism. The gene order is conserved among the three species, except for OppA, duplicated in *B. japonicum and R. palustris*. In this tree, gene names are colored according to the following code: *M. massiliensis,* blue; α-proteobacteria, yellow; β-proteobacteria, red; γ-proteobacteria, green; and others, black. The trees were built using a maximum likelihood substitution model and midpoint rooting.

Similarity searches showed that 632 ORFs (17.1%) had a best match with water-living organisms from other clades, mainly α- and γ-proteobacteria (Table 1). This apparently high rate of laterally transferred genes was confirmed by phylogenetic analysis, which showed that at least 65% of those 632 ORFs had no phylogenetic affinity with genes from other β-proteobacteria. Furthermore, 236 of those ORFs belonged to a group of two or more consecutive genes with a best match in the same source organism, suggesting en-bloc gene transfer (see Materials and Methods). Genomic organization and phylogenetic analyses of the oligopeptide permease operon OppABCDE in M. massiliensis (β-proteobacteria, mma1401–1405), Bradyrhizobium japonicum (α-proteobacteria), and *Rhodopseudomonas palustris* (α-proteobacteria) exemplified this en-bloc gene transfer (Figure 4). Putative transferred genes belonged mainly to E-COG (amino acid transport and metabolism), K-COG (transcription), O-COG (posttranslational modification, protein turnover, and chaperones) and P-COG (inorganic ion transport and metabolism). Putative transferred genes that could not be assigned to COG categories encoded sensors, transporters, and TonB-dependent receptors, including siderophore receptors.

**Table 1:**
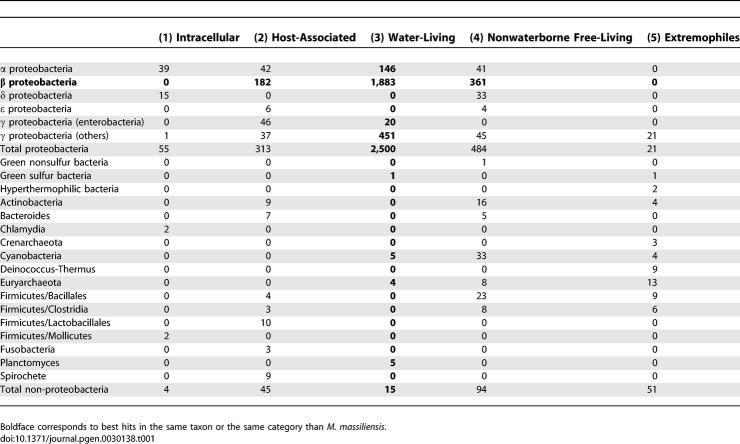
Repartition of Best Hits of 3,697 M. massiliensis ORFS against Proteins in the Kegg Database, According to Phylogeny and Environmental Categories of Organisms

### Heavy-Metal and Antibiotic Resistance

**Figure 5:**
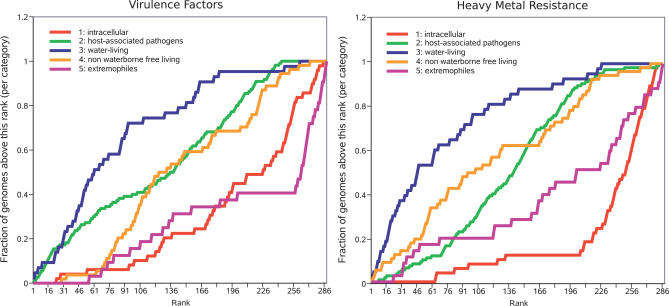
Prevalence of Virulence Factors and Heavy-Metal Resistance Genes in Water-Living Bacteria Organisms are ranked according to the number of hits to the virulence factor database or the heavy-metal resistance database (Materials and Methods) they exhibited per unit length of genome size. For each rank, the fraction of organisms in this category with the same rank or below is plotted. The lifestyle categories are the same as those in Figure 3. In this representation, we show that the genomes of water-living organisms tend to rank higher, showing a higher density of virulence factors and heavy-metal resistance genes.

The M. massiliensis genome encodes an unexpected capacity for heavy-metal and metalloid resistance, with some genes clustered in resistance island selfish operons [14]. The M. massiliensis genome harbors two copies of the two-component system for copper resistance (CopABCD, plus CopR sensor and CopS kinase, mma1721–1726, and mma0793–0798) instead of one copy, as is found in its nearest evolutionary neighbors. The cadmium/cobalt/zinc resistance system identified here is also found in Pseudomonas spp. and R. solanacearum. Cointegrate resolution proteins S and T are located upstream and downstream of the mercuric resistance operon MerEDACPTBR (mma1747–1754), very similar to a pHCM1 plasmid–borne copy found in Salmonella typhi strain CT18 and to the mercuric resistance operon of N. europaea and R. eutropha. Chromate resistance is provided by the operon ChrAB (mma3047/3048), an isolated copy of ChrA (mma1941), plus two sets of two ChrA half-sized homologs (mma0176/0177 and mma1187/1188). The tellurium resistance gene TerC is present in two copies (mma0089/0686). Arsenic resistance is provided by an ArsRBH operon (mma2629–2631), an arsenite transport protein ArsB (mma0720), and two putative arsenate reductases ArsC (mma2071/3429). The M. massiliensis genome exhibits a complete potassium transport system KdpABCDE (mma1819–1823), as is reported in P. aeruginosa, *Chromobacterium violaceum,* and Escherichia coli. Further analyses indicated that the density of heavy-metal resistance genes was higher in water-living bacteria than in any other category of organisms (Figure 5).

M. massiliensis also possesses class A and class C beta-lactamases (mma0118/0443/0705/1284/2306) and streptomycin kinase (mma1889). Consistently, M. massiliensis was found to be resistant to penicillin (minimal inhibitory concentration of >32 mg/L) and streptomycin (minimal inhibitory concentration of >500 mg/L), but susceptible to other aminoglycosides.

### Virulence Factors and Iron Metabolism

Similarity search analysis against a set of Swiss-Prot [15] entries related to bacterial virulence identified 155 putative virulence factors in M. massiliensis. When this analysis was extended to 287 available complete proteomes, M. massiliensis ranked 44 in the number of hits. After normalization based on the genome size, M. massiliensis ranked 10, amidst important human pathogens (Table S3). Two-component systems represent 30% of the putative virulence factors. Such systems, consisting of a sensor histidine kinase and a response regulator, have been identified in major pathogens [16]. We also identified 15 autotransporter proteins, usually used by gram-negative bacteria to deliver large-size virulence factors [17]. M. massiliensis is well-equipped for iron uptake and metabolism. It encodes 16 FecR copies (Fe^2+^-dicitrate sensor, membrane components) that are always associated with an RpoE ECF subfamily sigma factor (FecI) and a supplementary gene. This structure resembles that reported for N. europaea [18], with 20 FecIR gene tandems. Among these supplementary genes, 12 encode 820-amino acid siderophore receptors and four encode uncharacterized giant proteins Ugp1–4 (mma1391/1922/2361/2368), the largest genes in the *M. massiliensis* genome. However, we found no evidence of a complete siderophore biosynthesis pathway. We identified three HmsHRF (mma2647–2649) components of the hemin storage system and, among several iron uptake–related proteins, the ferrous iron transport proteins FeoA and FeoB (mma1835/1836). We also identified two nearby genes encoding Bfr1 and Bfr2 ferritin (mma0361/0362), probably arising from a recent duplication event. As for iron-uptake regulation, probing the M. massiliensis genome with the 19-bp consensus Fur box GATAATGAT(A/T)ATCATTATC from *E. coli* resulted in a total of 26 hits (allowing up to four mismatches), 16 of which were located upstream of iron-related ORFs. The M. massiliensis genome also encodes a complete type IV pilus operon, suggesting its capacity to acquire additional resistance markers or virulence factors.

## Discussion

### Filterability and Resistance to Water Threats

The ability of prokaryotes to escape filtration has been questioned based on theoretical grounds [19,20], but filterable water-living β-proteobacteria, *Actinobacteria* and *Spirochaetae* were cultured and observed by culture-independent methods [4,21,22]. Bacteria benefit from their small size in several ways. In agreement with previous observations that small size protects water-living bacteria against predation by bacteriovorous nanoflagellates [23,24] and amoebas [25], M. massiliensis is not killed by amoebas (see Materials and Methods). Moreover, amoebas have been shown to favor positive selection of virulence factors in Legionella pneumophila, *P*. *aeruginosa,* and other water-living bacteria [26]. The same virulence factors may be used to resist the bactericidal effect of human macrophages and, in several cases, resistance to amoebal killing predicts pathogenicity in mammals [25]. Another benefit of small size is that the surface-to-volume ratio is reduced, enhancing nutrient uptake. As for *M. massiliensis,* the small volume of its SCV is indicative of an ultimately reduced metabolic activity coupled to a large surface-to-volume ratio that optimizes exchanges with nutrient-poor, purified hospital water, pending an encounter with a more favorable medium in which the less favorable surface-to-volume ratio of LCV becomes sustainable. Most water-living oligotrophic bacteria tend to have a small volume of <0.1 μm^3^, probably reflecting similar constraints [27]. The cell of M. massiliensis SCV, although its dimensions are comparable to that of Pelagibacter ubique, contains a genome that is three times larger [28]. With a DNA compaction value of 650 mg/ml, typical of bacterial nucleoids [29], the nucleoid of the M. massiliensis SCV may represent more than 60% of the total cell volume, further reducing the volume available to metabolic activity. The mechanisms governing bacterial cell shape and its relation to chromosome dynamics remain largely unknown. They involve bacterial cell wall and cytoskeleton components as well as penicillin binding proteins and membrane-bound determinants, all of which are found in M. massiliensis [30]. A homolog of histone H1, which modulates nucleoid size during the transition between the two developmental forms (small elementary body form and large reticulate body form) of Chlamydia trachomatis [31], is also found in M. massiliensis.

### Pooling Genes in the Water

**Figure 6:**
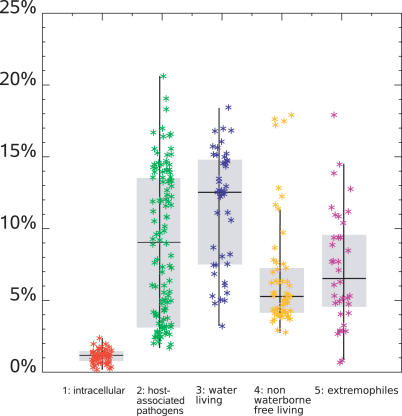
Distribution of Putative LGTs Organisms are colored according to lifestyle, which are the same as in Figure 3.

Lateral gene transfer (LGT) is thought to be a major source of evolution among bacterial communities [14]. Phylogenetic analysis of the 17% of M. massiliensis genes exhibiting a best match with water-living organisms from other clades was indicative of a high proportion of LGT. This prompted us to investigate the contribution of LGT in bacterial communities in various environments. Indeed, we found that LGT from distant clades varied among bacteria according to their lifestyle (Figure 6; Materials and Methods). Bacterial communities living in water exhibited the highest percentage of LGT when compared to other categories of organisms. Intracellular bacteria exemplified a radically opposite evolution strategy of limited exchanges among a limited number of organisms as exemplified for the intra-amoebal Rickettsia bellii [32]. Other large microbial communities exhibited an intermediate strategy, with less LGT for host-associated bacteria (13% ± 4% versus 9% ± 5%) and nonwater-living free organisms (13% ± 4% versus 5% ± 2 %). Metagenomic analyses of the gut flora, an example of a host-associated bacterial community, indicated restricted diversity in an otherwise enormous population of bacteria belonging to a few bacterial divisions [33,34]. These data suggest that water-living bacteria evolved with both genomic and functional convergences in order to thrive in their complex, ever-changing medium. Water is a privileged medium for exchanging DNA molecules, providing water-living bacteria with ample opportunity to acquire adaptive traits that are literally “floating around.”

### Water-Living Bacteria, a Reservoir for Virulence and Resistance?

M. massiliensis gene content is consistent with its resistance to water disinfection and its presence in hospital UPW. A unique genomic island encodes resistance to the heavy-metal ions and metalloids used for water disinfection [35]. M. massiliensis also encodes 23 copies of RpoE and genome-wide scattered heavy-metal control systems involved in metal resistance regulation, as shown in E. coli and Pseudomonas putida [36,37]. Dense regulation was previously interpreted as enabling rapid adaptation to ever-changing environmental conditions for free-living environmental organisms [13,38]. Further analyses indicated that, among environmental organisms, water-living bacteria contained more heavy-metal resistance genes (Figure 5), suggesting that these organisms may act as a source for their transfer to other bacteria. M. massiliensis encodes several antibiotic resistance genes and is resistant to penicillin and streptomycin. Likewise, the emergence of plasmid-mediated resistance to quinolones in Enterobacteriaceae, an important group of pathogens, has been recently traced to the water-living inhabitant Shewanella algae [39]. These data highlight that water-living bacteria, including important nosocomial pathogens such as P. aeruginosa and Acinetobacter spp. [40], could serve as a reservoir for genes encoding antibiotic degradation. M. massiliensis is unexpectedly well-equipped for iron uptake and regulation, with its large set of *Fur* genes. Iron uptake is a key for bacterial virulence [41]. Hence, patients with iron overload have a higher risk of infection with environmental organisms [41]. The presence of siderophore receptors without a siderophore biosynthesis pathway suggests that M. massiliensis might utilize siderophores produced by other environmental organisms. Iron is an important growth factor for pathogenic bacteria as it is crucial for microbial replication, electron transport, glycolysis, DNA synthesis, and defense against toxic reactive oxygen intermediates [41]. Moreover, the M. massiliensis genome encodes other known virulence factors such as hemolysin and type IV and type V secretion systems. This organism confirms the observation that the density of putative virulence factor genes is higher among water-living bacteria than those in any other category (Figure 5).

### Conclusions

*M. massiliensis,* a newly discovered waterborne motile bacterium, passes through filters, survives in water and appears capable of detoxifying its environment. Its resistance to amoebal predators is consistent with the presence of many virulence factors in its genome. It appears well adapted to its environment and endowed with a high exchange rate with bacterial water communities, 17% of its genes putatively originating from lateral transfer, including antibiotic resistance and heavy-metal resistance genes. M. massiliensis illustrates a new threat, by its capacity to acquire and promote the exchange of virulence factors and resistance genes among present and future nosocomial agents.

## Materials and Methods

### Isolation of strains and growth conditions.

UPW hospital samples were incubated at 30 °C on trypticase casein soy and R2A agar (Bio-Rad Laboratories, http://www.bio-rad.com/). Cells were examined for morphology following Gram staining and phase-contrast microscopy. The presence of flagella was assessed by depositing bacteria on formvar film, and staining with a 0.33% solution of uranyl acetate before examination on a Philips Morgagni 268 D electron microscope (FEI Company, http://www.fei.com). Cell size and volume was determined in stationary-stage organisms in UPW based on epifluorescence and electron microscopy data. For epifluorescence microscopy, cells were stained with lipophilic marker FM-464 (Invitrogen, http://www.invitrogen.com) and DAPI (Invitrogen) and observed with an epifluorescence microscope. Precise measurements (*n* = 20 organisms) were difficult to obtain due to fluorescence blurry edge of cells and the small cell size. We then calculated cell volume using the formula: volume = 4/3π*ab* ^2^ where “a” designs the half-length and “b” the maximum half-width and the surface using the formula: surface = 2π*b* ^2^(1 + (*a*/*b*) arcsin(*e*)/*e*), *e = √(1 − b* ^2^/*a* ^2^) after electron microscopy observation of 50 microorganisms. For filtration experiments, the isolate calibrated at 10^8^ cfu/ml into dialysis fluid was filtered through a 0.45-μm filter or 0.20-μm filter (Corning, http://www.corning.com/). M. massiliensis type strain Marseille was deposited into the Collection de l'Institut Pasteur and the Culture Collection University of Göteborg.

### Resistance to amoebas.

An Acanthamoeba polyphaga strain, Linc AP-1 (provided by T. J. Rowbotham, Leeds Public Health Laboratory, Leeds, United Kingdom), was grown at 30 °C in a 150-cm^2^ cell-culture flask with 30 ml of peptone yeast extract glucose broth. When the concentration reached 10^5^/ml, as determined by counting in a Nageotte cell with trypan blue, the amoebae were harvested and pelleted by centrifugation. The supernatant was removed, and the amoebae were resuspended in 50 ml of Page's amoebic saline (PAS). Centrifugation and resuspension in PAS were repeated twice. After the last centrifugation, amoebae distributed in 10-mL culture flasks were centrifuged for 30 min at 2,500 rpm in the presence of 3 × 10^10^ cfu of M. massiliensis and incubated at 35 °C in a 2.5% to 5% CO_2_ atmosphere for 7 d. Every day, the microplate was gently shaken in order to suspend amoebas, and 100 μl of the suspension was used for cytocentrifugation. Slides were Giemsa stained. The experiment was done twice. No intra-amoebal organism was detected during the 6-d observation period when parallel engulfment of E. coli and Staphylococcus aureus used as positive control organisms demonstrated that the amoebas were still able to prey on bacteria.

### Shotgun of M. massiliensis genome, sequencing strategy, and annotation.

DNA was extracted by incubation with 1% SDS and RNAseI at 37 °C for 2 h followed by an overnight lytic treatment with Proteinase K at 37 °C. After three phenol–chloroform extractions and ethanol precipitation, DNA was resuspended in TE pH 7.5. No plasmid was observed after loading DNA extraction on a 0.6% agarose gel in 1× TBE. Following mechanical shearing, two shotgun genomic libraries were constructed of 4- and 6-kb inserts in pCDNA2.1 (Invitrogen). A third library was constructed using mini BACs. About 40 μg of genomic DNA was partially digested by Sau3A endonuclease (New England Biolabs, http://www.neb.com/) and 10–25 kb DNA fragments were ligated into dephosphorylated BamHI-digested pBBC vector. The quality of the library was validated by analysis of 96 clones digested by NotI (New England Biolabs). Sequencing was carried out using Big Dye 3.1 (Applera, http://www.applera.com/) terminator chemistry on an automated capillary ABI 3700 sequencer (Applera). The three libraries yielded respectively 17,497, 23,250, and 10,436 sequencing reads from both ends of inserts, corresponding to a 9-fold coverage of the genome. Sequences were analyzed and assembled into contigs using Phred, Phrap, and Consed softwares [42,43] taking all sequences into account. Finishing included 568 directed sequencing reactions analyzed on an ABI3100 sequencer (Applera). The final assembly contained 99.95% of positions with a Phred/Phrap score above 40. An initial set of protein-coding genes was detected using self-training Markov models [44] and careful examination of intergenic regions to rescue additional ORFs. ORFs were then validated and annotated by sequence similarity using Blastp [45] against the nonredundant protein database from the National Center for Biotechnology Information (NCBI) and the Kegg protein database [11], and by profile detection using RPSblast [45] and the COG database [12]. Genes encoding tRNA were identified with tRNAscan-SE [46] and other RNAs were located using Blastn [45].

### Phylogenomic analysis.

We retrieved protein sequence data for bacterial genomes in the Kegg database [11] and COG data from NCBI [12]. Each complete proteome was compared to the COG profile database using the RPSblast program [45]. A significance score was determined for each COG so that any sequence not used to build the COG profile scored below this score. Any proteome was thus converted in a COG vector whose components represent the presence (1) or absence (0) of a significant match to each COG. Correlation of COG vectors was computed and a distance was defined between any pair of organism (o_i_ and o_j_) as distance = 1 – correlation(o_i,_o_j_). The matrix of distances was converted into a tree using the neighbor program (UPGMA algorithm) from the Phylip package [47]. Similar analyses can be performed using only a subset of the COGs, or only a subset of the organisms. The whole tree-drawing procedure is fully automatic and readers are welcome to perform their analyses on our server (http://www.igs.cnrs-mrs.fr/CogTree/cogtree.cgi).

### LGT analysis.

All genes from completely sequenced bacteria, classified according to their lifestyle, were mutually compared with the Blat program [48]. For each organism, a gene was regarded as resulting from a LGT event when its best hit was to an organism in the same lifestyle category, but from another clade. The list of M. massiliensis genes with a best match in category 3 organisms (water-living organisms) from another clade was further studied in the following way. We counted how often successive genes in this list had a best match to the same target organism. This resulted in 236 genes. The same analysis applied to the randomized list of genes resulted in 71 (standard deviation = 11) genes, indicating that consecutive genes tend to show phylogenetic affinity with the same source organism, suggesting en-bloc gene acquisitions.

### Virulence factors and heavy-metal resistance.

Sequence entries with the keyword “virulence” were extracted from the Swiss-Prot database [15] to build a dataset of 1,055 virulence-related genes. Likewise, all putative heavy-metal resistance genes (90) were extracted from the genome of M. massiliensis. Using the blastall program [45] (*e*-value = 1.0 e−10), we counted the number of hits between those sets of sequences and the available complete prokaryotic proteomes in the Kegg database [11] plus the predicted proteome of M. massiliensis. Bacteria were ranked according to the number of hits per unit length of genome size, and for each environmental category of organism, we plotted the number of bacteria above a given rank (Figure 5).

## Supporting Information

Figure S1 Comparative Purine Metabolism in R. solanaceraum and M. massiliensis
Enzymes present in R. solanaceraum are represented by green rectangles. A red mark indicates enzymes absent from *M. massiliensis.* A green mark indicates enzymes present in M. massiliensis but absent from *R. solanaceraum.* We gratefully acknowledge the use of metabolic pathway drawings from the Kegg database (http://www.genome.jp/kegg/).
(103 KB PDF)

Figure S2 COG-Based Phylogenomic Representation of M. massiliensis
Trees for C-COGs (energy production and conversion), D-COG (cell division and chromosome partitioning), and I-COG (lipid metabolism) show clustering of organisms according to lifestyle rather than to the 16s rDNA–based phylogeny. The position in the tree of M. massiliensis is indicated by a red triangle. Data for other COGs is available at http://www.igs.cnrs-mrs.fr/CogTree/cogtree.cgi.
(5.3 MB PDF)

Table S1 Main Phenotypic Characteristics of M. massiliensis gen. nov. sp. nov. Growth and hemolysis at different temperatures were determined in tubes of nutrient broth (Difco, http://www.bd.com/) and Columbia agar with 5% sheep blood (Bio Mérieux, http://www.biomerieux.com/) incubated for 3 d in water baths set at 4 °C, 22 °C, 25 °C, 30 °C, 35 °C, 37 °C, and 42 °C. Growth was further tested at 30 °C on trypticase soy agar, chocolate agar (Bio Mérieux), Mac Conkey agar (Bio-Rad Laboratories), and BCYE agar (Oxoid, http://www.oxoid.com/). Oxidase activity was detected using a dimethyl-para-phenylenediamine oxalate disk (Bio-Rad Laboratories). Catalase activity was detected by emulsifying a colony in 3% hydrogen peroxide and checking for the presence of microscopic bubbles. A set of 40 physiological characteristics were tested by inoculation of API 20 E and API 20 NE strips according to the recommendations of the supplier (BioMérieux) and incubation at 30 °C for 48 h. Strip tests were done three times. The API 20 NE strip tested for any reduction of nitrates, indole production, urease activity, glucose acidification, arginine dihydrolase activity, hydrolysis of gelatin and esculin, beta-galactosidase activity, and assimilation of glucose, arabinose, mannose, mannitol, N-acetyl-glucosamine, maltose, gluconate, caprate, adipate, malate, citrate, and phenyl-acetate. As interpretation of arginine dihydrolase and gelatinase activities on this strip was difficult, detection of these activities were later performed on ADH-ODC-LDC broth (Bio-Rad Laboratories) and nutrient gelatin (Oxoid) respectively, according to the manufacturers' instructions and incubated at 30 °C for 7 d. H_2_S production was tested using sodium thiosulfate substrate (BioMérieux). Antibiotic susceptibility testing was performed using the disk diffusion method [49]. The plates were incubated at 30 °C and read 72 h later. E. coli and Enterococcus faecalis were used as controls.
(20 KB DOC)

Table S2 Transport-Related Genes Found in the Genome of M. massiliensis
Genes are classified using data from TransportDB (http://www.membranetransport.org) and the Transporter Classification Database (http://www.tcdb.org). Genes that most likely work together are grouped as functional units.
(204 KB DOC)

Table S3 Occurrences of “Virulence”-Like Genes in Prokaryotic Genomes Table is sorted according to the number of hits per megabase of genome.
(418 KB DOC)

### Accession Numbers

The NCBI GenBank accession number for the complete genome sequence of Minibacterium massiliensis is CP000269.

The M. massiliensis type strain Marseille was deposited into the Collection de l'Institut Pasteur (http://www.crbip.pasteur.fr/) under accession number CIP 107820^T^ and the Culture Collection University of Göteborg (http://www.ccug.se/) under accession number CCUG 50593^T^.

The American Type Culture Collection (http://www.atcc.org) accession numbers for the E. coli and E. faecalis controls are 25922 and 29212, respectively.

## Abbreviations

COG: cluster of orthologous groups
LGT: lateral gene transfer
LCV: large-cell variant
SCV: small-cell variant
UPW: ultrapure water
