# Supplementary material for: Genome Analysis of Minibacterium massiliensis Highlights the Convergent Evolution of Water-Living Bacteria
Source: PLoS Genet. 2007 Aug 24;3(8):e138. doi: 10.1371/journal.pgen.0030138 (PMC1950954; doi:10.1371/journal.pgen.0030138)
Supplement: Supplementary file 1 [file pgen.0030138.sg001.pdf]

Purine Metabolism in *Ralstonia solanaceraum*

Enzyme absent from *Minibacterium massiliensis*

Enzyme present in *Minibacterium massiliensis*

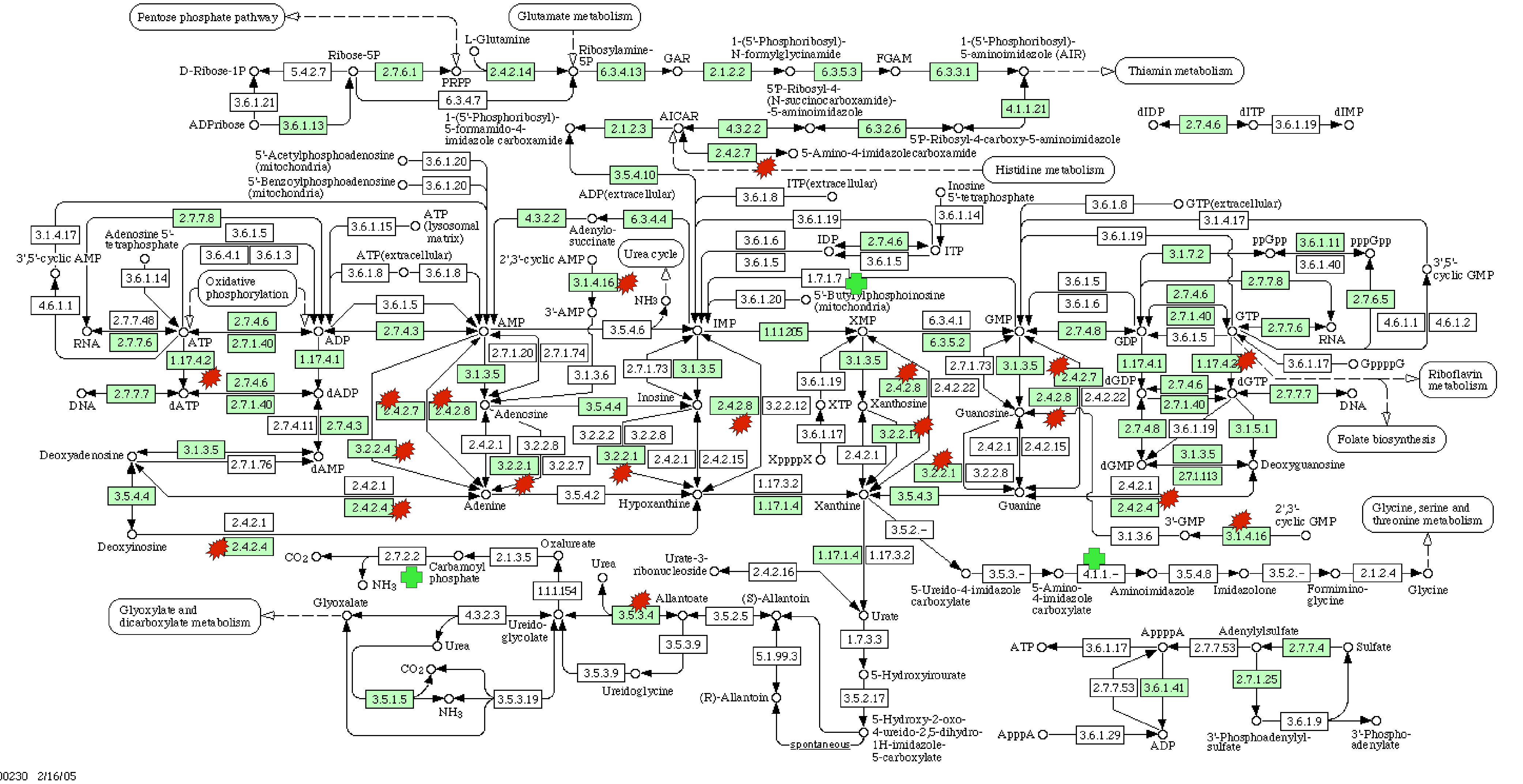

Figure S1
