# Supplementary material for: Genome Analysis of Minibacterium massiliensis Highlights the Convergent Evolution of Water-Living Bacteria
Source: PLoS Genet. 2007 Aug 24;3(8):e138. doi: 10.1371/journal.pgen.0030138 (PMC1950954; doi:10.1371/journal.pgen.0030138)
Supplement: Supplementary file 2 [file pgen.0030138.sg002.pdf]

Cog C tree (Energy production and conversion)

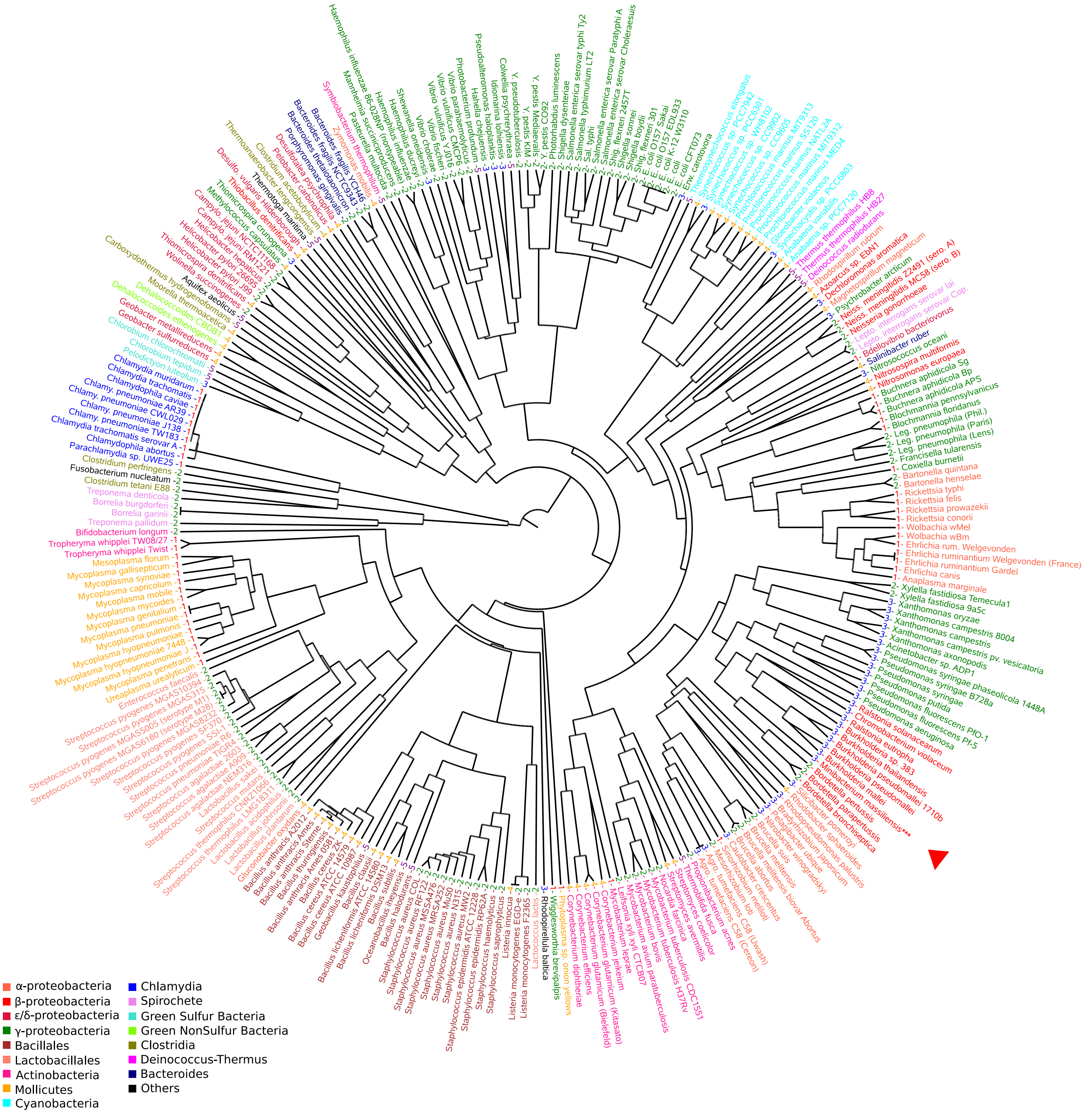

Figure S2:1/3

Cog D tree (Cell division and chromosome partitionning)

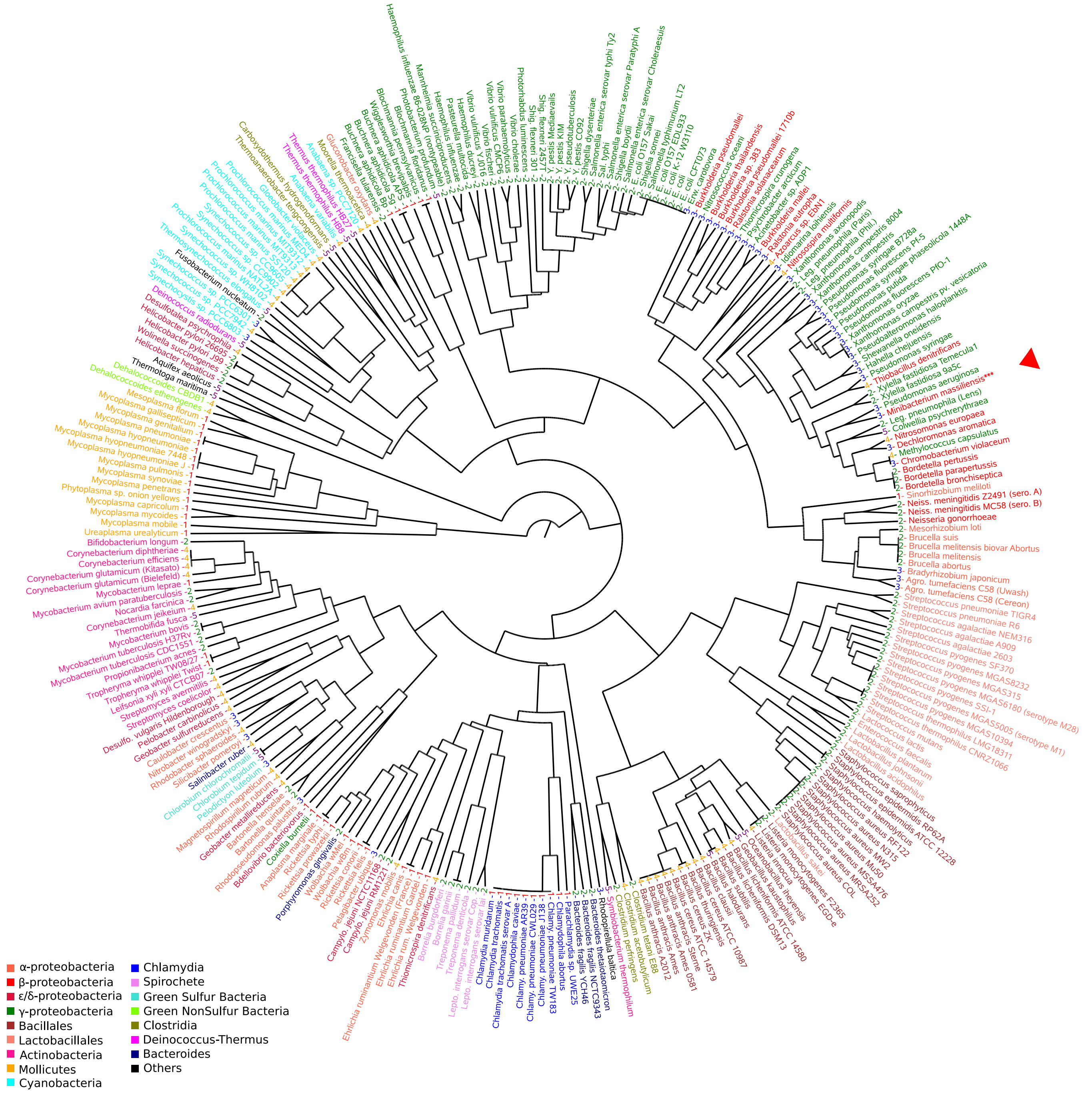

Figure S2:2/3

Cog I tree (Lipid metabolism)

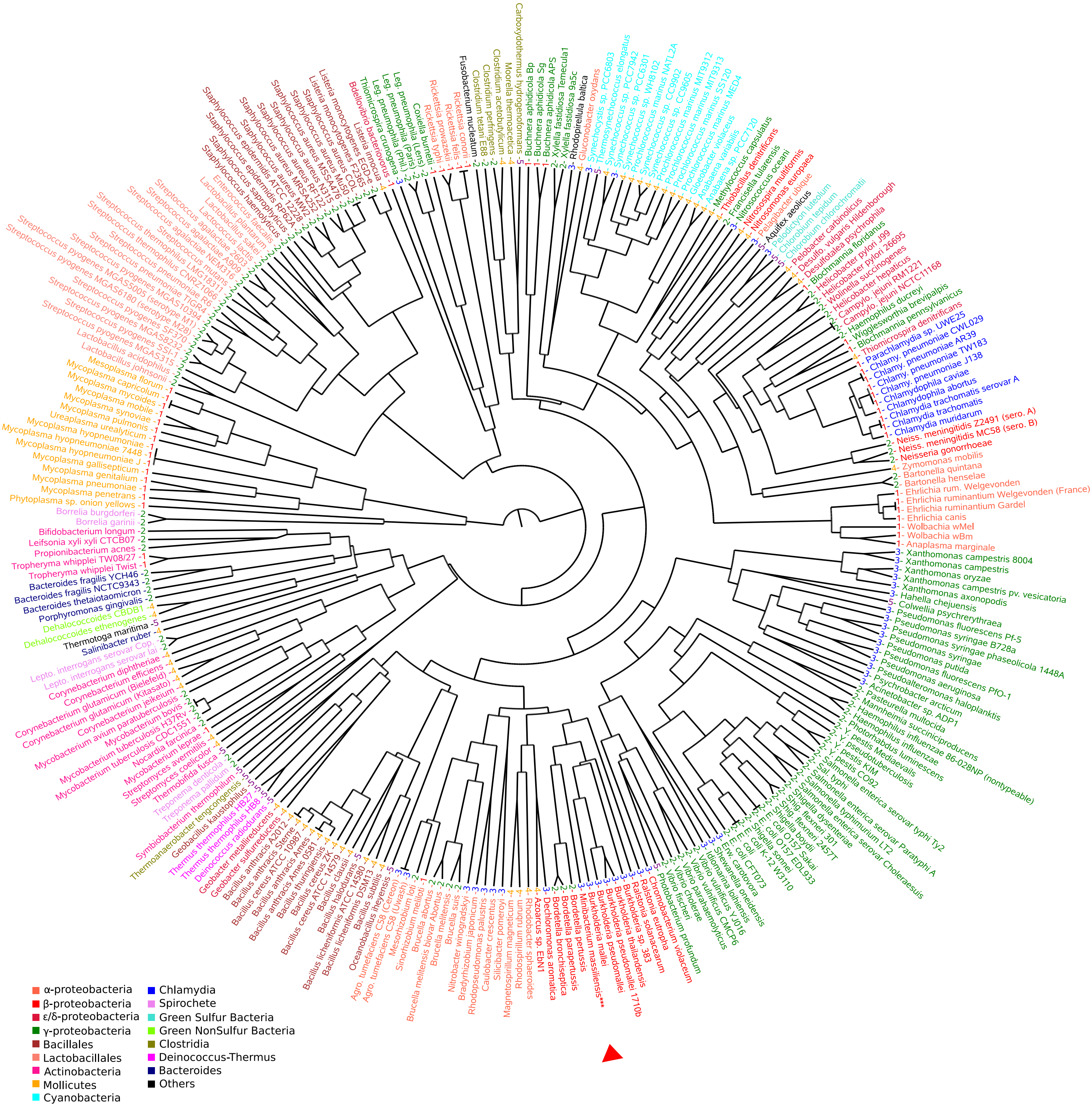

Figure S2:3/3
