# Supplementary material for: Genome Analysis of Minibacterium massiliensis Highlights the Convergent Evolution of Water-Living Bacteria
Source: PLoS Genet. 2007 Aug 24;3(8):e138. doi: 10.1371/journal.pgen.0030138 (PMC1950954; doi:10.1371/journal.pgen.0030138)
Supplement: Supplementary file 3 [file pgen.0030138.st001.doc]

Supplementary Table S1: Main phenotypic characteristics of *M. massiliensis* gen. nov. sp. nov.

Characteristic	Result

________________________________________________________________

Cell morphology	Gram-negative, short rods

Cell diameter (nm)	235-550

Cell width (nm)	645-1,300

Motility	+

Oxydase	+

Catalase	+

Urease	-

Nitrate reduction	+

at 4°C	+

at 22°C	+

Growth	at 25°C	+

at 30°C	+

at 35°C	-

at 42°C	-

Utilization of (number of days for positivity)

DL - lactase	2d

Glutarate	2d

DL-glycerate	2d

L-malate	4d

Fumarate	4d

Succinate	4d

L-glutamate	4d

L-alamine	4d

Xylitol	6d

Adonitol	6d

Susceptibility to

Penicillin	-

Amoxicillin	+

Streptomycine	-

Gentamycine	+

Ciprofloxacine	+

Fosfomycine	-

Vancomycine	-

DNA G + C content (mol %)	54.22

________________________________________________________________
