# Supplementary material for: Genome Analysis of Minibacterium massiliensis Highlights the Convergent Evolution of Water-Living Bacteria
Source: PLoS Genet. 2007 Aug 24;3(8):e138. doi: 10.1371/journal.pgen.0030138 (PMC1950954; doi:10.1371/journal.pgen.0030138)
Supplement: Supplementary file 4 [file pgen.0030138.st002.doc]

**Supplementary Table S2:** Transport related genes found in the genome of *Minibacterium massiliensis*

| **Transporter Family** | **TC number** | **# orfs: 432** | **# systems: 260** | Gene name | **Function** |
| --- | --- | --- | --- | --- | --- |
| ATP-Dependent |  |  |  |  |  |
| ABC family (ions) | 3.A.1 | 3 | 1 | mma0812-4(cysUWA) | sulfate ABC transporter |
|  |  | 4 | 1 | mma0739-42(ssuADCB) | sulfonate/nitrate transport system |
|  |  | 1 | 1 | mma1021(ssuA) | sulfonate ABC transporter, periplasmic sulfonate-binding protein SsuA |
|  |  | 2 | 1 | mma1659-60 | ABC-type nitrate/sulfonate/bicarbonate transport system |
|  |  | 1 | 1 | mma1496(modA) | molybdenum ABC transporter, periplasmic molybdenum-binding protein |
|  |  | 2 | 1 | mma1963-4(x,cysT) | ABC-type tungstate transport system |
|  |  | 4 | 1 | mma2288-91(pstBCS) | ABC-type phosphate transport system |
|  |  | 3 | 1 | mma3040-2(phnCDE) | phosphonate ABC-type transport system |
|  |  | 1 | 1 | mma2345(phnD) | phosphonate transport system substrate-binding protein |
|  |  | 3 | 1 | mma2483-5(tauCBA) | ABC-type sulfonate/nitrate transport system |
|  |  | 3 | 1 | mma2590-2(cysPUW) | ABC-type sulfate/thiosulfate transport system |
|  |  | 4 | 1 | mma2616-9 | ABC-type nitrate/sulfonate/bicarbonate transport system |
|  |  | 3 | 1 | mma3084-6 | ABC-type nitrate/sulfonate/bicarbonate transport system |
|  |  | 1 | 1 | mma3465 | ABC-type nitrate/sulfonate/bicarbonate transport systems, periplasmic components |
|  |  | 1 | 1 | mma3649(tupB) | ABC-type tungstate transport system, permease component |
|  |  | 4 | 1 | mma3534-7 | ABC-type nitrate/sulfonate/bicarbonate transport system |
|  |  | 3 | 1 | mma3656-8(modCBA) | ABC-type molybdate transport system |
|  |  | 3 | 1 | mma0480-2(nasFED) | ABC-type nitrate transport system |
|  |  | 1 | 1 | mma1070 | putative component of ABC-type phosphonate transport system |
| ABC family (amino-acids) | 3.A.1 | 4 | 1 | mma0033-6 | ABC-type polar amino acid transport system |
|  |  | 5 | 1 | mma0656-60(livKHMGF) | ABC-type branched-chain amino acid transport system |
|  |  | 1 | 1 | mma0665 | ABC-type branched-chain amino acid transport systems, periplasmic component |
|  |  | 5 | 1 | mma0778-82(livKHMGF) | ABC-type branched-chain amino acid transport systems, periplasmic component |
|  |  | 5 | 1 | mma0095-9(livFGMHK) | ABC-type branched-chain amino acid transport system |
|  |  | 5 | 1 | mma1806-10(livKHMGF) | ABC-type branched-chain amino acid transport system |
|  |  | 5 | 1 | mma1549-53(livKHMGF) | ABC-type branched-chain amino acid transport system permease component |
|  |  | 1 | 1 | mma2301 | ABC-type branched-chain amino acid transport systems, periplasmic component |
|  |  | 4 | 1 | mma2545-8(livFGMH) | ABC-type branched-chain amino acid transport system |
|  |  | 1 | 1 | mma3184 | ABC-type branched-chain amino acid transport systems, periplasmic component |
|  |  | 1 | 1 | mma3266 | ABC-type branched-chain amino acid transport systems, periplasmic component |
|  |  | 4 | 1 | mma3519-22(dppDCBA) | ABC-type dipeptide/oligopeptide/nickel transport system |
|  |  | 4 | 1 | mma0279-82(gltIJKL) | ABC-type glutamate/aspartate transport system permease protein |
|  |  | 5 | 1 | mma1401-5(oppABCDE) | ABC-type oligopeptide transport system |
| ABC family (drug) | 3.A.1 | 2 | 1 | mma3292-3 | ABC-type multidrug transport system |
|  |  | 2 | 1 | mma1620-1 | ABC-type multidrug transport system |
|  |  | 2 | 1 | mma0253-4 | ABC-type multidrug transport system |
|  |  | 2 | 1 | mma3553-4 | ABC-type multidrug transport system |
|  |  | 1 | 1 | mma1065 | putative macrolide ABC efllux protein |
|  |  | 1 | 1 | mma0535(msbA) | ABC-type multidrug transport system, ATPase and permease components |
|  |  | 1 | 1 | mma1194 | ABC-type multidrug transport system, ATPase and permease components |
| ABC family (iron related) | 3.A.1 | 2 | 1 | mma0727-8(fbpBC) | iron(III) ABC-type transport system |
|  |  | 1 | 1 | mma2249(futA) | ABC-type Fe3+ transport system, periplasmic component |
|  |  | 3 | 1 | mma0944-6(ccmCBA) | heme ABC export system |
|  |  | 1 | 1 | mma0277(atm1) | ABC-type transport system involved in Fe-S cluster assembly, permease and ATPase components |
|  |  | 2 | 1 | mma1093-4 | ABC-type cobalamin/Fe3+-siderophores transport system |
| ABC family (others) | 3.A.1 | 1 | 1 | mma3374 | ABC-type transport system involved in cytochrome c biogenesis, permease component |
|  |  | 2 | 1 | mma3382-3(nodJI) | ABC-2 type transport system permease protein |
|  |  | 4 | 1 | mma3103-6(potDCBA) | spermidine/putrescine transport system |
|  |  | 2 | 1 | mma3140-1(ftsEX) | cell division related ABC-type transport system |
|  |  | 2 | 1 | mma2015-6(cydDC) | ABC-type transport system involved in cytochrome bd biosynthesis |
|  |  | 2 | 1 | mma1265-6(lolED) | ABC-type transport system, involved in lipoprotein release |
|  |  | 1 | 1 | mma1342 | Predicted ABC-type transport system involved in lysophospholipase L1 biosynthesis, permease component |
|  |  | 1 | 1 | mma1537 | Predicted ABC-type transport system involved in lysophospholipase L1 biosynthesis, ATPase component |
| ABC family (uncharacterized) | 3.A.1 | 2 | 1 | mma3207-8 | ABC-type uncharacterized transport system |
|  |  | 5 | 1 | mma3240-4(birA,x,x,x,x) | ABC-type uncharacterized transport system |
|  |  | 5 | 1 | mma3295-9(x,vacJ,x,x,x) | ABC-type uncharacterized transport system |
|  |  | 1 | 1 | mma0271 | ABC-type uncharacterized transport system, permease and ATPase components |
|  |  | 2 | 1 | mma3350-1 | ABC-type uncharacterized transport system |
|  |  | 2 | 1 | mma0259-60 | ABC-type uncharacterized transport system |
|  |  | 1 | 1 | mma2984 | ABC-type uncharacterized transport system, permease component |
|  |  | 2 | 1 | mma0264-5 | ABC-type uncharacterized transport system |
|  |  | 4 | 1 | mma3562-5 | ABC-type uncharacterized transport system |
|  |  | 1 | 1 | mma2522(uup) | probable ATP-binding ABC transporter protein |
|  |  | 2 | 1 | mma1829-30 | ABC-type uncharacterized transport system |
|  |  | 1 | 1 | mma1670 | Uncharacterized ABC-type transport system, periplasmic component/surface lipoprotein |
|  |  | 3 | 1 | mma1677-9 | ABC-type uncharacterized transport systems |
|  |  | 1 | 1 | mma3254 | ABC-type transport system, ATPase and permease components |
|  |  | 1 | 1 | mma2074 | ATPase components of ABC transporters with duplicated ATPase domains |
|  |  | 1 | 1 | mma2001 | ATPase components of ABC transporters with duplicated ATPase domains |
|  |  | 4 | 1 | mma1204-7 | ABC-type uncharacterized transport system |
|  |  | 1 | 1 | mma1100 | ABC-type transport system, periplasmic component |
| F-ATPase family | 3.A.2 | 8 | 1 | mma3626-33 (atpCDGAHFEB) | F-type H+-transporting ATPase |
| H+-PPase family | 3.A.10 | 1 | 1 | mma2575(rrpP) | H+ translocating pyrophosphate synthase |
| P-ATPase family | 3.A.3 | 4 | 4 | mma1629, mma1714, mma1796(zntA1), mma3525(zntA2) | cation-translocating P-type ATPase |
|  |  | 3 | 1 | mma1821-3 (kdpCBA) | multicomponent K+ transporting ATPase |
|  |  | 1 | 1 | mma2556(mgtC) | Mg2+ transport ATPase |
|  |  | 1 | 1 | mma1768 | mgtC family protein |
| Ion Channels |  |  |  |  |  |
| ClC family | 1.A.11 | 1 | 1 | mma1585(eriC) | chloride channel protein eriC |
| Hsp70 family | 1.A.33 | 1 | 1 | mma0196(mreB) | rod shape-determining protein MreB |
|  |  | 1 | 1 | mma1256(hscA) | molecular chaperone HscA (Hsc66) |
|  |  | 1 | 1 | mma2883(dnaK) | molecular chaperone DnaK (Hsp70) |
| MIP family | 1.A.8 | 1 | 1 | mma2544(aqpZ) | aquaporin Z, transmembrane water channel (MIP family) |
| MIT family | 1.A.35 | 2 | 2 | mma0084(corA1), mma2960(corA2) | magnesium and cobalt transport protein, metal ion transporter, MIT family |
|  |  | 1 | 1 | mma2655 | putative cation transporter (MIT family) |
| MscL family | 1.A.22 | 1 | 1 | mma3272(mscL) | large conductance mechanosensitive channel, MscL family |
| MscS family | 1.A.23 | 4 | 4 | mma2429, mma2431,mma2568,mma3580 | mechanosensitive ion channel protein (mscS family) |
| UAC family | 1.A.29 | 1 | 1 | mma1895 | putative transporter protein (UAC family) |
| VIC family | 1.A.1 | 2 | 2 | mma0736(tas), mma3546 | probable oxidoreductase, aldo/keto reductase family |
| Phosphotransferase System (PTS) |  |  |  |  |  |
| GPTS family | N.A. | 2 | 1 | mma0174-5(pstHI) | sugar transport PTS system |
| SSPTS family | N.A. | 2 | 2 | mma1800(ptsN1), mma3118(ptsN2) | PTS system, nitrogen regulatory IIA component |
|  |  | 1 | 1 | mma0173 | putative IIa component of sugar transport PTS system |
|  |  | 1 | 1 | mma2041(ppsa) | pyruvate,water dikinase |
| Secondary Transporter |  |  |  |  |  |
| AAE family | 2.A.81 | 2 | 2 | mma1082, mma1083 | alanine/aspartate exchanger |
| Amt family | 2.A.49 | 3 | 3 | mma0170(amtB1), mma0589(amtB2), mma2510(amtB3) | ammonium transporter, Amt family |
| ArsB family | 2.A.45 | 2 | 2 | mma0720(arsB1), mma2630(arsB2) | arsenite transport protein arsB |
| BCCT family | 2.A.15 | 1 | 1 | mma0207(betT) | high-affinity choline transporter |
| BenE family | 2.A.46 | 1 | 1 | mma2006(benE) | benzoate transport protein |
| CDF family | 2.A.4 | 2 | 2 | mma1767(czcD1), mma1826(czcD2) | cobalt-zinc-cadmium resistance protein czcD |
| CHR family | 2.A.51 | 4 | 2 | mma0176-7, mma1187-8 | chromate transporter chrA half-sized homolgs |
|  |  | 2 | 2 | mma1941(chrA1), mma3047(chrA2) | chromate transporter chrA |
| CPA1 family | 2.A.36 | 1 | 1 | mma2011(yjcE) | monovalent cation:H+ antiporter, CPA1 family |
| CPA2 family | 2.A.37 | 3 | 3 | mma0955(kefB1), mma1936(kefB2), mma3346(kefB3) | glutathione-regulated potassium efflux system protein kefB |
|  |  | 1 | 1 | mma2039(hflC) | monovalent cation:H+ antiporter, CP2 family |
|  |  | 1 | 1 | mma2606 | probable NA/H+ antiporter |
| CPA3 family | 2.A.63 | 6 | 1 | mma3256-61(phaACDEFG) | multicomponent K+:H+ antiporter |
| DAACS family | 2.A.23 | 1 | 1 | mma1996(dctA) | dicarboxylate/amino acid:cation (Na+ or H+) symporter, DAACS family |
| DMT family | 2.A.7 | 1 | 1 | mma0078(sugE) | transporter of the DMT superfamily, sugE homolog |
|  |  | 2 | 2 | mma2583(rarD1), mma3532(rarD2) | transporter of the DMT superfamily, Chloramphenicol-Sensitivity Protein (rarD family) |
|  |  | 1 | 1 | mma1733(cnrT) | transporter of the DMT superfamily, probable cobalt-nickel-resistance system transmembrane protein |
|  |  | 1 | 1 | mma3344 | transporter of the DMT superfamily, small multidrug resistance (SMR) family |
|  |  | 11 | 11 | mma0327, mma0404, mma0549, mma0605, mma0700, mma0730, mma1147, mma1579, mma1915, mma1937, mma2440 | transporter of the DMT superfamily |
| ESS family | 2.A.27 | 1 | 1 | mma1604(gltS) | glutamate:Na+ symporter, ESS family |
| KUP family | 2.A.72 | 1 | 1 | mma3533(kup) | KUP system potassium uptake protein |
| LctP family | 2.A.14 | 2 | 2 | mma3077(lldP1), mma3187(lldP2) | lactate transporter, lctP family |
| MFS family | 2.A.1 | 1 | 1 | mma2176(aas) | 2-acylglycerophosphoethanolamine acyltransferase |
|  |  | 1 | 1 | mma0184(ampG) | putative MFS transporter and signal transducer AmpG |
|  |  | 6 | 6 | mma2542(oxlT2), mma1456(oxlT1), mma3672(oxlT6), mma3473(oxlT4), mma2642(oxlT3), mma3508(oxlT5) | oxalate/formate antiporter, MFS superfamily |
|  |  | 2 | 2 | mma0886(shiA1), mma2392(shiA2) | Shikimate transporter shiA, MFS superfamily |
|  |  | 2 | 2 | mma3324(tcaB), mma2906(bcr) | drug resistance MFS transporter, Bcr/CflA family |
|  |  | 1 | 1 | mma1047(yeaN) | transporter of the MFS superfamily, similar to yeaN (e.coli) |
|  |  | 30 | 30 | mma0094, mma0188, mma0346, mma0573, mma0757, mma0871, mma1041, mma1524, mma1558, mma1590, mma1600, mma1602, mma1610, mma1657, mma1854, mma1867, mma1869, mma1993, mma2296, mma2316, mma2481, mma2633, mma2641, mma3095, mma3181, mma3253, mma3340, mma3432, mma3517, mma3668 | drug, monocarboxilate, etc.. |
| MOP family | 2.A.66 | 2 | 2 | mma0648, mma2267 | polysaccharide transport protein (MOP family) |
|  |  | 1 | 1 | mma2520(mviN) | MVIN, virulence factor (MOP family) |
| NCS1 family | 2.A.39 | 1 | 1 | mma1067 | cytosine/purine/uracil/thiamine/allantoin permease family protein (NCS1 family) |
| NCS2 family | 2.A.40 | 7 | 7 | mma0226(pbuX1), mma0512(pbuX2), mma1239(pbuX3), mma1241(pbuX4), mma2502(pbuX5), mma2508(pbuX6), mma3675(pbuX7) | xanthine permease (NSC2 family) |
|  |  | 1 | 1 | mma0508 | uracil/xanthine permease (NCS2 family) |
| NhaA family | 2.A.33 | 1 | 1 | mma1077(nhaA) | Na+:H+ antiporter, NhaA family |
| Oxa1 family | 2.A.9 | 1 | 1 | mma3694(yidC) | preprotein translocase YidC subunit |
| PiT family | 2.A.20 | 1 | 1 | mma1307(pitA) | inorganic phosphate transporter, PiT family |
| RhtB family | 2.A.76 | 1 | 1 | mma0569(rhtB) | Homoserine/homoserine lactone/beta-hydroxynorvaline efflux permease (rhtB family) |
|  |  | 1 | 1 | mma0699 | Uncharacterized rhtB family transporter |
| RND family | 2.A.6 | 3 | 1 | mma0245-7(exbBDD) | Biopolymer transport |
|  |  | 2 | 1 | mma0363-5(exbDB,tonB) | Biopolymer transport |
|  |  | 2 | 1 | mma2378-9(exbBD) | Biopolymer transport |
|  |  | 2 | 1 | mma2580-1(exbDB) | Biopolymer transport |
|  |  | 2 | 1 | mma2893-4(exbBD) | multidrug efflux system |
|  |  | 3 | 1 | mma0020-2(acrBA,tolC) | multidrug efflux system |
|  |  | 3 | 1 | mma0050-2(tolC.acrAB) | multidrug efflux system |
|  |  | 4 | 1 | mma2934-7(tolC,acrRAB) | multidrug efflux system |
|  |  | 2 | 1 | mma2332-3(x,acrA) | multidrug efflux system |
|  |  | 3 | 1 | mma1564-6(acrA,x) | multidrug efflux system |
|  |  | 3 | 1 | mma0352-4(yajC,secDF) | Preprotein translocase |
|  |  | 1 | 1 | mma0761 | Uncharacterized conserved domain protein |
|  |  | 3 | 1 | mma1024-6(x,x,ompR2) | cation or multidrug |
|  |  | 1 | 1 | mma1064 | multidrug efflux system |
|  |  | 3 | 1 | mma1122-4(tolC,emrAB) | multidrug efflux system |
|  |  | 3 | 1 | mma1681-3(tolC,yegON) | multidrug efflux system |
|  |  | 3 | 1 | mma1717-9(x,x,silA) | uncharacterized efflux pump |
|  |  | 3 | 1 | mma1730-2(x,czcBA) | cation efflux system |
|  |  | 3 | 1 | mma1769-71(czcA,glnB,czcB) | cation efflux system |
|  |  | 3 | 1 | mma1792-4(czcABC) | cation efflux system |
|  |  | 3 | 1 | mma0285-7(tolC,czcAB) | cation efflux system |
|  |  | 3 | 1 | mma0409-11(czcAB,x) | cation efflux system |
|  |  | 1 | 1 | mma3052-3(mexC,x) | multidrug efflux system |
| SSS family | 2.A.21 | 2 | 2 | mma0899, mma2866 | sodium/solute transporter SSS family |
| SulP family | 2.A.53 | 3 | 3 | mma0088 (hvsT), mma1109( sulP), mma2411(sul1) | sulfate permeases, sulP family |
|  |  | 2 | 2 | mma2596(cynT1), mma2880(cynT2) | carbonic anhydrase |
|  |  | 1 | 1 | mma0186 | putative sulfate transport related protein |
| Tat family | 2.A.64 | 2 | 2 | mma1267(tatD1), mma1982(tatD2) | Mg-dependent DNase |
|  |  | 3 | 1 | mma3277-9 (tatCBE) | sec-independent protein translocase proteins (tatC, tatB, tatE) |
| TDT family | 2.A.16 | 1 | 1 | mma2816(tehA) | tellurite resistance protein tehA |
| TRAP-T family | 2.A.56 | 6 | 2 | mma0125-7 (fcbT1lsp), mma1135-7(fcbTpls) | TRAP-type mannitol/chloroaromatic compound transport system, large permease component |
|  |  | 1 | 1 | mma0469(dctP) | C4-dicarboxylate-binding periplasmic protein |
|  |  | 1 | 1 | mma1075 | Uncharacterized conserved protein |
| Trk family | 2.A.38 | 2 | 1 | mma3593-4(trkGA) | Trk-type K+ transport systems, membrane component |
|  |  | 1 | 1 | mma2944(apaH) | bis(5'-nucleosyl)-tetraphosphatase (symmetrical) |
| TTT family | 2.A.80 | 18 | 6 | mma0101-3 (tctCBA1), mma0394-6(tctCBA2), mma0438-40(tctABC3), mma0470-2(tctCBA4), mma0816-7(tctAB5), mma0822(tctC5), mma1291-3(tctCBA6) | tripartite tricarboxylate transporter |
|  |  | 4 | 4 | mma1709(tctC7), mma1852(tctC8), mma2387(tctC9), mma2446(tctC10) | isolate tricarboxylate binding receptor (no permase components) |
| Unclassified |  |  |  |  |  |
| FeoB family | 9.A.8 | 1 | 1 | mma1835(feoB) | ferrous iron transport protein B |
| MerTP family | 9.A.2 | 1 | 1 | mma1752(merT) | putative mercuric transport protein |
|  |  | 1 | 1 | mma3528 | putative copper-binding protein |
| PbrT family | 9.A.17 | 1 | 1 | mma1853 | high affinity FE2+/Pb2+ permease |
| PnuC family | 9.A.4 | 1 | 1 | mma0047(pnuC) | nicotinamide mononucleotide transporter pnuC |
|  |  |  |  |  |  |
