# Supplementary material for: Genome Analysis of Minibacterium massiliensis Highlights the Convergent Evolution of Water-Living Bacteria
Source: PLoS Genet. 2007 Aug 24;3(8):e138. doi: 10.1371/journal.pgen.0030138 (PMC1950954; doi:10.1371/journal.pgen.0030138)
Supplement: Supplementary file 5 [file pgen.0030138.st003.doc]

**Supplementary Table S3:** Occurences of “virulence” like genes in prokaryotic genomes. The table is sorted according to the number of hits per megabase of genome.

| **rank** | **Number of Hits per Mbp** | **Number of Hits** | **Kegg id** | **Genome Size (bp)** | **Environ-mental category** | Classification | **Organism name** |
| --- | --- | --- | --- | --- | --- | --- | --- |
| 1 | 42.31 | 201 | cvi | 4,751,080 | 3 | β-proteobacteria | c.violaceum |
| 2 | 41.35 | 205 | stm | 4,951,371 | 2 | γ-proteobacteria | c.typhimurium |
| 3 | 41.34 | 259 | pae | 6,264,404 | 3 | γ-proteobacteria | p.aeruginosa |
| 4 | 39.64 | 192 | yps | 4,840,898 | 2 | γ-proteobacteria | y.pseudotuberculosis |
| 5 | 39.47 | 181 | spt | 4,585,229 | 2 | γ-proteobacteria | s.enterica_paratyphi |
| 6 | 39.32 | 177 | dar | 4,501,104 | 3 | β-proteobacteria | d.aromatica |
| 7 | 39.10 | 202 | vpa | 5,165,770 | 2 | γ-proteobacteria | v.parahaemolyticus |
| 8 | 39.03 | 193 | sec | 4,944,000 | 2 | γ-proteobacteria | s.enterica_choleraesuis |
| 9 | 38.40 | 184 | stt | 4,791,961 | 2 | γ-proteobacteria | s.typhi_ty2 |
| 10 | 37.84 | 155 |  | 4,110,251 | 3 | β–proteobacteria | m.massiliensis |
| 11 | 37.67 | 106 | sac | 2,813,862 | 2 | Bacillales | s.aureus_col |
| 12 | 37.67 | 208 | ece | 5,620,522 | 2 | γ-proteobacteria | e.coli_o157 |
| 13 | 37.27 | 179 | ypm | 4,803,217 | 2 | γ-proteobacteria | y.pestis_mediaevails |
| 14 | 37.03 | 179 | ype | 4,829,855 | 2 | γ-proteobacteria | y.pestis |
| 15 | 36.64 | 205 | ecs | 5,594,477 | 2 | γ-proteobacteria | e.coli_o157j |
| 16 | 36.52 | 103 | sam | 2,820,462 | 2 | Bacillales | s.aureus_mw2 |
| 17 | 36.24 | 175 | sfl | 4,828,821 | 2 | γ-proteobacteria | s.flexneri |
| 18 | 36.21 | 186 | sty | 5,133,713 | 2 | γ-proteobacteria | s.typhi |
| 19 | 36.21 | 103 | sau | 2,839,469 | 2 | Bacillales | s.aureus_n315 |
| 20 | 35.76 | 104 | sav | 2,903,636 | 2 | Bacillales | s.aureus_mu50 |
| 21 | 35.69 | 101 | sas | 2,820,454 | 2 | Bacillales | s.aureus_mssa476 |
| 22 | 35.34 | 255 | hch | 7,215,267 | 3 | γ-proteobacteria | h.chejuensis |
| 23 | 35.26 | 227 | pfo | 6,438,405 | 3 | γ-proteobacteria | p.fluorescens_pfo1 |
| 24 | 35.08 | 23 | buc | 655,725 | 1 | γ-proteobacteria | buchnera |
| 25 | 34.49 | 157 | sdy | 4,551,958 | 2 | γ-proteobacteria | s.dysenteriae |
| 26 | 34.35 | 243 | pfl | 7,074,893 | 3 | γ-proteobacteria | p.fluorescens |
| 27 | 34.33 | 173 | ssn | 5,039,661 | 2 | γ-proteobacteria | s.sonnei |
| 28 | 34.30 | 22 | bas | 641,454 | 1 | γ-proteobacteria | b.aphidicola_sg |
| 29 | 34.14 | 96 | neu | 2,812,094 | 4 | β-proteobacteria | n.europaea |
| 30 | 33.89 | 294 | bur | 8,676,277 | 3 | β-proteobacteria | burkholderia_383 |
| 31 | 33.81 | 206 | psb | 6,093,698 | 3 | γ-proteobacteria | p.syringae_b728a |
| 32 | 33.76 | 227 | bte | 6,723,972 | 3 | β-proteobacteria | b.thailandensis |
| 33 | 33.58 | 144 | vfi | 4,284,050 | 2 | γ-proteobacteria | v.fischeri |
| 34 | 33.34 | 97 | tbd | 2,909,809 | 4 | β-proteobacteria | t.denitrificans |
| 35 | 33.32 | 206 | ppu | 6,181,863 | 3 | γ-proteobacteria | p.putida |
| 36 | 33.18 | 91 | sab | 2,742,531 | 2 | Bacillales | s.aureus_rf122 |
| 37 | 33.07 | 96 | sar | 2,902,619 | 2 | Bacillales | s.aureus_mrsa252 |
| 38 | 32.98 | 153 | eco | 4,639,675 | 2 | γ-proteobacteria | e.coli |
| 39 | 32.69 | 172 | vvy | 5,260,086 | 2 | γ-proteobacteria | v.vulnificus_yj016 |
| 40 | 32.58 | 165 | eca | 5,064,019 | 3 | γ-proteobacteria | e.carotovora |
| 41 | 32.50 | 151 | ecj | 4,646,332 | 2 | γ-proteobacteria | e.coli_j |
| 42 | 32.48 | 131 | vch | 4,033,464 | 2 | γ-proteobacteria | v.cholerae |
| 43 | 32.01 | 232 | bps | 7,247,547 | 3 | β-proteobacteria | b.pseudomallei |
| 44 | 31.88 | 233 | bpm | 7,308,054 | 3 | β-proteobacteria | b.pseudomallei_1710b |
| 45 | 31.60 | 162 | vvu | 5,126,797 | 2 | γ-proteobacteria | v.vulnificus |
| 46 | 31.21 | 191 | psp | 6,112,448 | 3 | γ-proteobacteria | p.syringae_phaseolicola |
| 47 | 31.16 | 163 | ecc | 5,231,428 | 2 | γ-proteobacteria | e.coli_cft073 |
| 48 | 30.99 | 146 | ypk | 4,701,745 | 2 | γ-proteobacteria | y.pestis_kim |
| 49 | 30.63 | 178 | rso | 5,810,922 | 3 | β-proteobacteria | r.solanacearum |
| 50 | 30.39 | 117 | pha | 3,850,272 | 3 | γ-proteobacteria | p.haloplanktis |
| 51 | 30.27 | 198 | pst | 6,538,260 | 3 | γ-proteobacteria | p.syringae |
| 52 | 30.13 | 140 | sbo | 4,646,520 | 2 | γ-proteobacteria | s.boydii |
| 53 | 29.94 | 85 | ilo | 2,839,318 | 3 | γ-proteobacteria | i.loihiensis |
| 54 | 29.87 | 21 | wbr | 703,004 | 1 | γ-proteobacteria | w.brevipalpis |
| 55 | 29.82 | 174 | bma | 5,835,527 | 3 | β-proteobacteria | b.mallei |
| 56 | 29.75 | 80 | tte | 2,689,445 | 5 | Clostridia | t.tengcongensis |
| 57 | 29.63 | 76 | sep | 2,564,615 | 2 | Bacillales | s.epidermidis |
| 58 | 29.59 | 158 | bbr | 5,339,179 | 2 | β-proteobacteria | b.bronchiseptica |
| 59 | 29.38 | 118 | ccr | 4,016,947 | 3 | α-proteobacteria | c.crescentus |
| 60 | 29.09 | 77 | ser | 2,643,840 | 2 | Bacillales | s.epidermidis_rp62a |
| 61 | 29.07 | 211 | reu | 7,255,290 | 3 | β-proteobacteria | r.eutropha |
| 62 | 28.74 | 55 | hit | 1,913,428 | 2 | γ-proteobacteria | h.influenzae_nt |
| 63 | 28.70 | 207 | ana | 7,211,789 | 4 | Cyanobacteria | anabaena |
| 64 | 28.63 | 117 | bpe | 4,086,189 | 2 | β-proteobacteria | b.pertussis |
| 65 | 28.49 | 136 | bpa | 4,773,551 | 2 | β-proteobacteria | b.parapertussis |
| 66 | 28.41 | 52 | hin | 1,830,138 | 2 | γ-proteobacteria | h.influenzae |
| 67 | 28.41 | 150 | xac | 5,274,174 | 3 | γ-proteobacteria | x.axonopodis |
| 68 | 28.29 | 152 | cps | 5,373,180 | 5 | γ-proteobacteria | c.psychrerythraea |
| 69 | 28.23 | 119 | bsu | 4,214,630 | 4 | Bacillales | b.subtilis |
| 70 | 28.23 | 153 | xcv | 5,420,152 | 3 | γ-proteobacteria | x.campestris_vesicatoria |
| 71 | 27.94 | 118 | bld | 4,222,645 | 4 | Bacillales | b.licheniformis_dsm13 |
| 72 | 27.91 | 63 | pmu | 2,257,487 | 2 | γ-proteobacteria | p.multocida |
| 73 | 27.87 | 197 | ava | 7,068,601 | 4 | Cyanobacteria | a.variabilis |
| 74 | 27.82 | 100 | gka | 3,592,666 | 5 | Bacillales | g.kaustophilus |
| 75 | 27.79 | 100 | aci | 3,598,621 | 3 | γ-proteobacteria | acinetobacter_adp1 |
| 76 | 27.71 | 117 | bli | 4,222,334 | 4 | Bacillales | b.licheniformis |
| 77 | 27.60 | 157 | plu | 5,688,987 | 2 | γ-proteobacteria | p.luminescens |
| 78 | 27.58 | 114 | cac | 4,132,880 | 4 | Clostridia | c.acetobutylicum |
| 79 | 27.47 | 128 | gvi | 4,659,019 | 4 | Cyanobacteria | g.violaceus |
| 80 | 27.47 | 71 | ssp | 2,577,899 | 2 | Bacillales | s.saprophyticus |
| 81 | 27.44 | 176 | ppr | 6,403,280 | 5 | γ-proteobacteria | p.profundum |
| 82 | 27.27 | 145 | btk | 5,314,794 | 4 | Bacillales | b.thuringiensis |
| 83 | 26.96 | 124 | sfx | 4,599,354 | 2 | γ-proteobacteria | s.flexneri_2457t |
| 84 | 26.88 | 146 | bce | 5,427,083 | 4 | Bacillales | b.cereus |
| 85 | 26.82 | 72 | sha | 2,685,015 | 2 | Bacillales | s.haemolyticus |
| 86 | 26.79 | 136 | xcc | 5,076,188 | 3 | γ-proteobacteria | x.campestris |
| 87 | 26.77 | 65 | tcx | 2,427,734 | 3 | γ-proteobacteria | t.crunogena |
| 88 | 26.61 | 137 | xcb | 5,148,708 | 3 | γ-proteobacteria | x.campestris_b |
| 89 | 26.15 | 77 | lmo | 2,944,528 | 2 | Bacillales | l.monocytogenes |
| 90 | 26.14 | 105 | gme | 4,011,182 | 4 | δ-proteobacteria | g.metallireducens |
| 91 | 26.08 | 148 | atu | 5,674,064 | 3 | α-proteobacteria | a.tumefaciens |
| 92 | 25.96 | 141 | bca | 5,432,652 | 4 | Bacillales | b.cereus_atcc10987 |
| 93 | 25.92 | 133 | son | 5,131,416 | 3 | γ-proteobacteria | s.oneidensis |
| 94 | 25.91 | 147 | atc | 5,673,465 | 3 | α-proteobacteria | a.tumefaciens_c |
| 95 | 25.90 | 27 | ctr | 1,042,519 | 1 | Chlamydia | c.trachomatis |
| 96 | 25.89 | 94 | oih | 3,630,528 | 5 | Bacillales | o.iheyensis |
| 97 | 25.87 | 68 | mta | 2,628,784 | 4 | Clostridia | m.thermoacetica |
| 98 | 25.82 | 75 | lmf | 2,905,187 | 2 | Bacillales | l.monocytogenes_f2365 |
| 99 | 25.77 | 53 | zmo | 2,056,416 | 4 | α-proteobacteria | z.mobilis |
| 100 | 25.67 | 27 | cta | 1,051,969 | 1 | Chlamydia | c.trachomatis_a |
| 101 | 25.41 | 140 | bar | 5,503,926 | 4 | Bacillales | b.anthracis_ames0581 |
| 102 | 25.33 | 82 | nmu | 3,234,309 | 4 | β-proteobacteria | n.multiformis |
| 103 | 25.32 | 135 | baa | 5,370,060 | 4 | Bacillales | b.anthracis_a2012 |
| 104 | 25.31 | 43 | hdu | 1,698,955 | 2 | γ-proteobacteria | h.ducreyi |
| 105 | 25.10 | 92 | pca | 3,665,893 | 4 | δ-proteobacteria | p.carbinolicus |
| 106 | 24.98 | 60 | chy | 2,401,520 | 5 | Clostridia | c.hydrogenoformans |
| 107 | 24.65 | 93 | dvu | 3,773,159 | 4 | δ-proteobacteria | d.vulgaris |
| 108 | 24.48 | 128 | bat | 5,228,663 | 4 | Bacillales | b.anthracis_sterne |
| 109 | 24.45 | 32 | pub | 1,308,759 | 3 | α-proteobacteria | p.ubique |
| 110 | 24.44 | 52 | sak | 2,127,839 | 2 | Lactobacillales | s.agalactiae_a909 |
| 111 | 24.35 | 15 | bab | 618,379 | 1 | γ-proteobacteria | b.aphidicola_bp |
| 112 | 24.26 | 75 | cpe | 3,085,740 | 2 | Clostridia | c.perfringens |
| 113 | 24.25 | 107 | rru | 4,406,557 | 4 | α-proteobacteria | r.rubrum |
| 114 | 23.97 | 53 | san | 2,211,485 | 2 | Lactobacillales | s.agalactiae_nem316 |
| 115 | 23.96 | 140 | bcz | 5,843,235 | 4 | Bacillales | b.cereus_zk |
| 116 | 23.91 | 125 | ban | 5,227,293 | 4 | Bacillales | b.anthracis |
| 117 | 23.80 | 100 | bha | 4,202,352 | 5 | Bacillales | b.halodurans |
| 118 | 23.77 | 55 | msu | 2,314,078 | 2 | γ-proteobacteria | m.succiniciproducens |
| 119 | 23.60 | 90 | gsu | 3,814,139 | 4 | δ-proteobacteria | g.sulfurreducens |
| 120 | 23.55 | 117 | mag | 4,967,148 | 4 | α-proteobacteria | m.magneticum |
| 121 | 23.53 | 89 | bba | 3,782,950 | 1 | δ-proteobacteria | b.bacteriovorus |
| 122 | 23.37 | 77 | bme | 3,294,931 | 2 | α-proteobacteria | b.melitensis |
| 123 | 23.22 | 49 | wsu | 2,110,355 | 2 | ε-proteobacteria | w.succinogenes |
| 124 | 23.17 | 51 | tdn | 2,201,561 | 4 | ε-proteobacteria | t.denitrificans_atcc33889 |
| 125 | 23.01 | 154 | sme | 6,691,694 | 1 | α-proteobacteria | s.meliloti |
| 126 | 22.89 | 50 | nma | 2,184,406 | 2 | β-proteobacteria | n.meningitidis_a |
| 127 | 22.74 | 18 | bpn | 791,654 | 1 | γ-proteobacteria | b.pennsylvanicus |
| 128 | 22.68 | 48 | ttj | 2,116,056 | 5 | Deinococcus-Thermus | t.thermophilus_hb8 |
| 129 | 22.68 | 16 | bfl | 705,557 | 1 | γ-proteobacteria | b.floridanus |
| 130 | 22.67 | 112 | xoo | 4,941,439 | 3 | γ-proteobacteria | x.oryzae |
| 131 | 22.66 | 43 | spb | 1,897,573 | 2 | Lactobacillales | s.pyogenes_mgas6180 |
| 132 | 22.63 | 107 | eba | 4,727,255 | 4 | β-proteobacteria | azoarcus_ebn1 |
| 133 | 22.61 | 57 | xft | 2,521,148 | 2 | γ-proteobacteria | x.fastidiosa_t |
| 134 | 22.56 | 48 | tth | 2,127,482 | 5 | Deinococcus-Thermus | t.thermophilus |
| 135 | 22.44 | 51 | nme | 2,272,360 | 2 | β-proteobacteria | n.meningitidis |
| 136 | 22.31 | 96 | bcl | 4,303,871 | 4 | Bacillales | b.clausii |
| 137 | 22.21 | 24 | cmu | 1,080,451 | 1 | Chlamydia | c.muridarum |
| 138 | 22.15 | 79 | sth | 3,566,135 | 5 | Actinobacteria | s.thermophilum |
| 139 | 22.11 | 42 | spa | 1,899,877 | 2 | Lactobacillales | s.pyogenes_mgas10394 |
| 140 | 22.10 | 42 | spg | 1,900,521 | 2 | Lactobacillales | s.pyogenes_m3 |
| 141 | 21.91 | 72 | bmb | 3,286,445 | 2 | α-proteobacteria | b.abortus |
| 142 | 21.89 | 60 | xfa | 2,731,750 | 2 | γ-proteobacteria | x.fastidiosa |
| 143 | 21.80 | 77 | noc | 3,522,111 | 3 | γ-proteobacteria | n.oceani |
| 144 | 21.79 | 72 | mca | 3,304,561 | 4 | γ-proteobacteria | m.capsulatus |
| 145 | 21.76 | 47 | sag | 2,160,267 | 2 | Lactobacillales | s.agalactiae |
| 146 | 21.72 | 72 | bms | 3,315,175 | 2 | α-proteobacteria | b.suis |
| 147 | 21.64 | 41 | sps | 1,894,275 | 2 | Lactobacillales | s.pyogenes_ssi1 |
| 148 | 21.60 | 67 | lin | 3,093,113 | 4 | Bacillales | l.innocua |
| 149 | 21.54 | 96 | rsp | 4,451,915 | 4 | α-proteobacteria | r.sphaeroides |
| 150 | 21.35 | 70 | bmf | 3,278,307 | 2 | α-proteobacteria | b.melitensis_abortus |
| 151 | 21.27 | 76 | syn | 3,573,470 | 3 | Cyanobacteria | synechocystis |
| 152 | 21.21 | 39 | spz | 1,838,554 | 2 | Lactobacillales | s.pyogenes_mgas5005 |
| 153 | 21.11 | 40 | spm | 1,895,017 | 2 | Lactobacillales | s.pyogenes_m18 |
| 154 | 20.90 | 71 | lpn | 3,397,754 | 2 | γ-proteobacteria | l.pneumophila |
| 155 | 20.84 | 60 | ctc | 2,873,333 | 2 | Clostridia | c.tetani |
| 156 | 20.82 | 54 | tel | 2,593,857 | 5 | Cyanobacteria | t.elongatus |
| 157 | 20.71 | 40 | bhe | 1,931,047 | 2 | α-proteobacteria | b.henselae |
| 158 | 20.70 | 113 | rpa | 5,459,213 | 3 | α-proteobacteria | r.palustris |
| 159 | 20.65 | 95 | sil | 4,601,053 | 3 | α-proteobacteria | s.pomeroyi |
| 160 | 20.64 | 42 | cbu | 2,032,674 | 1 | γ-proteobacteria | c.burnetii |
| 161 | 20.51 | 38 | spy | 1,852,441 | 2 | Lactobacillales | s.pyogenes |
| 162 | 20.36 | 44 | spn | 2,160,842 | 2 | Lactobacillales | s.pneumoniae |
| 163 | 20.32 | 185 | bja | 9,105,828 | 3 | α-proteobacteria | b.japonicum |
| 164 | 20.28 | 69 | nwi | 3,402,093 | 3 | α-proteobacteria | n.winogradskyi |
| 165 | 20.26 | 69 | lpf | 3,405,519 | 2 | γ-proteobacteria | l.pneumophila_lens |
| 166 | 20.14 | 153 | mlo | 7,596,297 | 2 | α-proteobacteria | m.loti |
| 167 | 20.03 | 55 | syf | 2,742,269 | 4 | Cyanobacteria | synechococcus_pcc7942 |
| 168 | 19.81 | 72 | lpp | 3,635,495 | 2 | γ-proteobacteria | l.pneumophila_paris |
| 169 | 19.60 | 31 | bqu | 1,581,384 | 2 | α-proteobacteria | b.quintana |
| 170 | 19.52 | 31 | rfe | 1,587,240 | 1 | α-proteobacteria | r.felis |
| 171 | 19.49 | 32 | cje | 1,641,481 | 2 | ε-proteobacteria | c.jejuni |
| 172 | 19.47 | 23 | cca | 1,181,356 | 1 | Chlamydia | c.caviae |
| 173 | 19.37 | 71 | dps | 3,659,634 | 4 | δ-proteobacteria | d.psychrophila |
| 174 | 19.22 | 22 | cab | 1,144,377 | 1 | Chlamydia | c.abortus |
| 175 | 19.16 | 56 | gox | 2,922,384 | 4 | α-proteobacteria | g.oxydans |
| 176 | 19.07 | 174 | sma | 9,119,895 | 4 | Actinobacteria | s.avermitilis |
| 177 | 18.92 | 24 | rco | 1,268,755 | 1 | α-proteobacteria | r.conorii |
| 178 | 18.92 | 51 | syc | 2,696,255 | 3 | Cyanobacteria | synechococcus_pcc6301 |
| 179 | 18.89 | 21 | rpr | 1,111,523 | 1 | α-proteobacteria | r.prowazekii |
| 180 | 18.87 | 171 | sco | 9,054,847 | 4 | Actinobacteria | s.coelicolor |
| 181 | 18.56 | 33 | cjr | 1,777,831 | 2 | ε-proteobacteria | c.jejuni_rm1221 |
| 182 | 18.56 | 37 | lac | 1,993,564 | 2 | Lactobacillales | l.acidophilus |
| 183 | 18.49 | 49 | par | 2,650,701 | 3 | γ-proteobacteria | p.arcticum |
| 184 | 18.25 | 60 | dra | 3,284,156 | 5 | Deinococcus-Thermus | d.radiodurans |
| 185 | 18.18 | 43 | lla | 2,365,589 | 2 | Lactobacillales | l.lactis |
| 186 | 18.11 | 39 | ngo | 2,153,922 | 2 | β-proteobacteria | n.gonorrhoeae |
| 187 | 18.07 | 36 | ljo | 1,992,676 | 2 | Lactobacillales | l.johnsonii |
| 188 | 18.04 | 34 | lsa | 1,884,661 | 2 | Lactobacillales | l.sakei |
| 189 | 17.99 | 20 | rty | 1,111,496 | 1 | α-proteobacteria | r.typhi |
| 190 | 17.96 | 34 | ftu | 1,892,819 | 2 | γ-proteobacteria | f.tularensis |
| 191 | 17.95 | 22 | cpt | 1,225,935 | 1 | Chlamydia | c.pneumoniae_tw183 |
| 192 | 17.94 | 83 | lic | 4,627,366 | 2 | Spirochete | l.interrogans_copenhageni |
| 193 | 17.94 | 22 | cpj | 1,226,565 | 1 | Chlamydia | c.pneumoniae_j138 |
| 194 | 17.94 | 39 | fnu | 2,174,500 | 2 | Fusobacteria | f.nucleatum |
| 195 | 17.91 | 84 | lil | 4,691,184 | 2 | Spirochete | l.interrogans |
| 196 | 17.89 | 22 | cpa | 1,229,853 | 1 | Chlamydia | c.pneumoniae_ar39 |
| 197 | 17.88 | 22 | cpn | 1,230,230 | 1 | Chlamydia | c.pneumoniae |
| 198 | 17.85 | 60 | efa | 3,359,974 | 2 | Lactobacillales | e.faecalis |
| 199 | 17.66 | 36 | spr | 2,038,615 | 2 | Lactobacillales | s.pneumoniae_r6 |
| 200 | 17.39 | 29 | hpy | 1,667,867 | 2 | ε-proteobacteria | h.pylori |
| 201 | 17.30 | 63 | tfu | 3,642,249 | 5 | Actinobacteria | t.fusca |
| 202 | 17.25 | 31 | stl | 1,796,846 | 2 | Lactobacillales | s.thermophilus_lmg |
| 203 | 17.11 | 91 | bfr | 5,310,990 | 2 | Bacteroides | b.fragilis |
| 204 | 17.00 | 38 | sye | 2,234,828 | 4 | Cyanobacteria | synechococcus_cc9902 |
| 205 | 16.97 | 89 | bfs | 5,241,700 | 2 | Bacteroides | b.fragilis_nctc9343 |
| 206 | 16.74 | 34 | smu | 2,030,921 | 2 | Lactobacillales | s.mutans |
| 207 | 16.70 | 30 | stc | 1,796,226 | 2 | Lactobacillales | s.thermophilus_cnrz |
| 208 | 16.70 | 19 | tpa | 1,138,011 | 2 | Spirochete | t.pallidum |
| 209 | 16.20 | 15 | tws | 925,938 | 1 | Actinobacteria | t.whipplei_s |
| 210 | 16.18 | 15 | twh | 927,303 | 1 | Actinobacteria | t.whipplei |
| 211 | 16.15 | 58 | sru | 3,587,328 | 4 | Bacteroides | s.ruber |
| 212 | 16.11 | 54 | lpl | 3,348,625 | 2 | Lactobacillales | l.plantarum |
| 213 | 16.02 | 39 | syw | 2,434,428 | 4 | Cyanobacteria | synechococcus_wh8102 |
| 214 | 15.79 | 37 | pgi | 2,343,476 | 2 | Bacteroides | p.gingivalis |
| 215 | 15.71 | 39 | cjk | 2,476,822 | 4 | Actinobacteria | c.jeikeium |
| 216 | 15.56 | 28 | hhe | 1,799,146 | 2 | ε-proteobacteria | h.hepaticus |
| 217 | 15.48 | 40 | lxx | 2,584,158 | 2 | Actinobacteria | l.xyli |
| 218 | 15.21 | 25 | hpj | 1,643,831 | 2 | ε-proteobacteria | h.pylori_j99 |
| 219 | 15.13 | 15 | bga | 986,914 | 2 | Spirochete | b.garinii |
| 220 | 15.08 | 25 | pmm | 1,657,990 | 4 | Cyanobacteria | p.marinus_med4 |
| 221 | 14.93 | 49 | cgb | 3,282,708 | 4 | Actinobacteria | c.glutamicum_b |
| 222 | 14.81 | 49 | cgl | 3,309,401 | 4 | Actinobacteria | c.glutamicum |
| 223 | 14.74 | 37 | syd | 2,510,659 | 4 | Cyanobacteria | synechococcus_cc9605 |
| 224 | 14.63 | 25 | pmi | 1,709,204 | 4 | Cyanobacteria | p.marinus_mit9312 |
| 225 | 14.11 | 26 | pmn | 1,842,899 | 4 | Cyanobacteria | p.marinus_natl2a |
| 226 | 14.08 | 34 | pcu | 2,414,465 | 1 | Chlamydia | parachlamydia_uwe25 |
| 227 | 14.08 | 68 | mpa | 4,829,781 | 2 | Actinobacteria | m.avium_paratuberculosis |
| 228 | 13.86 | 14 | mcp | 1,010,023 | 1 | Mollicutes | m.capricolum |
| 229 | 13.69 | 33 | pmt | 2,410,873 | 4 | Cyanobacteria | p.marinus_mit9313 |
| 230 | 13.66 | 86 | nfa | 6,292,344 | 2 | Actinobacteria | n.farcinica |
| 231 | 13.65 | 86 | bth | 6,293,399 | 2 | Bacteroides | b.thetaiotaomicron |
| 232 | 13.63 | 60 | mtc | 4,403,837 | 2 | Actinobacteria | m.tuberculosis_cdc1551 |
| 233 | 13.60 | 60 | mtu | 4,411,532 | 2 | Actinobacteria | m.tuberculosis |
| 234 | 13.36 | 16 | ama | 1,197,687 | 1 | α-proteobacteria | a.marginale |
| 235 | 13.27 | 30 | blo | 2,260,266 | 2 | Actinobacteria | b.longum |
| 236 | 12.96 | 14 | wbm | 1,080,084 | 1 | α-proteobacteria | wolbachia_bma |
| 237 | 12.89 | 33 | pac | 2,560,265 | 2 | Actinobacteria | p.acnes |
| 238 | 12.89 | 56 | mbo | 4,345,492 | 2 | Actinobacteria | m.bovis |
| 239 | 12.62 | 16 | wol | 1,267,782 | 1 | α-proteobacteria | wolbachia_mel |
| 240 | 12.46 | 31 | cdi | 2,488,635 | 4 | Actinobacteria | c.diphtheriae |
| 241 | 12.39 | 39 | cef | 3,147,090 | 4 | Actinobacteria | c.efficiens |
| 242 | 11.96 | 34 | tde | 2,843,201 | 2 | Spirochete | t.denticola |
| 243 | 11.42 | 20 | pma | 1,751,080 | 4 | Cyanobacteria | p.marinus |
| 244 | 10.53 | 16 | bbu | 1,519,856 | 2 | Spirochete | b.burgdorferi |
| 245 | 10.34 | 6 | mge | 580,076 | 1 | Mollicutes | m.genitalium |
| 246 | 10.29 | 8 | mmo | 777,079 | 1 | Mollicutes | m.mobile |
| 247 | 10.22 | 73 | rba | 7,145,576 | 3 | Planctomyces | r.baltica |
| 248 | 9.34 | 9 | mpu | 963,879 | 1 | Mollicutes | m.pulmonis |
| 249 | 9.33 | 14 | erg | 1,499,920 | 1 | α-proteobacteria | e.ruminantium_gardel |
| 250 | 9.13 | 12 | ecn | 1,315,030 | 1 | α-proteobacteria | e.canis |
| 251 | 9.03 | 9 | mga | 996,422 | 1 | Mollicutes | m.gallisepticum |
| 252 | 8.79 | 36 | mma | 4,096,345 | 4 | Euryarchaeota | m.mazei |
| 253 | 8.59 | 13 | erw | 1,512,977 | 1 | α-proteobacteria | e.ruminantium_welgevonden |
| 254 | 8.57 | 7 | mpn | 816,394 | 1 | Mollicutes | m.pneumoniae |
| 255 | 8.57 | 13 | eru | 1,516,355 | 1 | α-proteobacteria | e.ruminantium |
| 256 | 7.98 | 6 | uur | 751,719 | 1 | Mollicutes | u.urealyticum |
| 257 | 7.96 | 26 | mle | 3,268,203 | 1 | Actinobacteria | m.leprae |
| 258 | 7.86 | 15 | pfu | 1,908,256 | 5 | Euryarchaeota | p.furiosus |
| 259 | 7.56 | 6 | mfl | 793,224 | 1 | Mollicutes | m.florum |
| 260 | 7.26 | 20 | nph | 2,749,696 | 5 | Euryarchaeota | n.pharaonis |
| 261 | 7.13 | 41 | mac | 5,751,492 | 4 | Euryarchaeota | m.acetivorans |
| 262 | 7.02 | 30 | hma | 4,274,642 | 3 | Euryarchaeota | h.marismortui |
| 263 | 6.70 | 14 | tko | 2,088,737 | 5 | Euryarchaeota | t.kodakaraensis |
| 264 | 6.61 | 17 | hal | 2,571,010 | 5 | Euryarchaeota | halobacterium |
| 265 | 6.60 | 8 | mmy | 1,211,703 | 1 | Mollicutes | m.mycoides |
| 266 | 6.56 | 32 | mba | 4,873,766 | 5 | Euryarchaeota | m.barkeri |
| 267 | 6.11 | 3 | neq | 490,885 | 5 | Nanoarchaeota | n.equitans |
| 268 | 5.75 | 10 | pho | 1,738,505 | 5 | Euryarchaeota | p.horikoshii |
| 269 | 5.71 | 10 | mth | 1,751,377 | 5 | Euryarchaeota | m.thermoautotrophicum |
| 270 | 5.65 | 10 | pab | 1,768,562 | 5 | Euryarchaeota | p.abyssi |
| 271 | 5.51 | 12 | afu | 2,178,400 | 5 | Euryarchaeota | a.fulgidus |
| 272 | 5.42 | 9 | mmp | 1,661,137 | 4 | Euryarchaeota | m.maripaludis |
| 273 | 5.15 | 7 | mpe | 1,358,633 | 1 | Mollicutes | m.penetrans |
| 274 | 5.00 | 4 | msy | 799,476 | 1 | Mollicutes | m.synoviae |
| 275 | 4.79 | 8 | ape | 1,669,696 | 5 | Crenarchaeota | a.pernix |
| 276 | 4.65 | 4 | poy | 860,631 | 1 | Mollicutes | phytoplasma_oy |
| 277 | 4.59 | 8 | mja | 1,739,927 | 5 | Euryarchaeota | m.jannaschii |
| 278 | 4.48 | 4 | mhy | 892,758 | 1 | Mollicutes | m.hyopneumoniae |
| 279 | 4.46 | 4 | mhj | 897,405 | 1 | Mollicutes | m.hyopneumoniae_j |
| 280 | 4.42 | 7 | tvo | 1,584,804 | 5 | Euryarchaeota | t.volcanium |
| 281 | 4.35 | 4 | mhp | 920,079 | 1 | Mollicutes | m.hyopneumoniae_7448 |
| 282 | 4.05 | 9 | pai | 2,222,430 | 5 | Crenarchaeota | p.aerophilum |
| 283 | 4.04 | 9 | sai | 2,225,959 | 5 | Crenarchaeota | s.acidocaldarius |
| 284 | 3.88 | 6 | pto | 1,545,895 | 5 | Euryarchaeota | p.torridus |
| 285 | 3.83 | 6 | tac | 1,564,906 | 1 | Euryarchaeota | t.acidophilum |
| 286 | 3.54 | 6 | mka | 1,694,969 | 5 | Euryarchaeota | m.kandleri |
| 287 | 3.34 | 10 | sso | 2,992,245 | 5 | Crenarchaeota | s.solfataricus |
| 288 | 2.97 | 8 | sto | 2,694,756 | 5 | Crenarchaeota | s.tokodaii |
